# Detection of Virus-Related Sequences Associated With Potential Etiologies of Hepatitis in Liver Tissue Samples From Rats, Mice, Shrews, and Bats

**DOI:** 10.3389/fmicb.2021.653873

**Published:** 2021-06-08

**Authors:** Wenqiao He, Yuhan Gao, Yuqi Wen, Xuemei Ke, Zejin Ou, Yongzhi Li, Huan He, Qing Chen

**Affiliations:** ^1^Department of Epidemiology, School of Public Health, Guangdong Provincial Key Laboratory of Tropical Disease Research, Southern Medical University, Guangzhou, China; ^2^Xiamen Center for Disease Control and Prevention, Xiamen, China

**Keywords:** viral community, liver, rat, shrew, bat, mouse

## Abstract

Hepatitis is a major global health concern. However, the etiology of 10–20% hepatitis cases remains unclear. Some hepatitis-associated viruses, like the hepatitis E virus, are zoonotic pathogens. Rats, shrews, and bats are reservoirs for many zoonotic pathogens. Therefore, understanding the virome in the liver of these animals is important for the investigation of the etiologies of hepatitis and monitoring the emerging zoonotic viruses. In this study, viral metagenomics and PCR methods were used to investigate viral communities in rats, mice, house shrews, and bats livers. Viral metagenomic analysis showed a diverse set of sequences in liver samples, comprising: sequences related to herpesviruses, orthomyxoviruses, anelloviruses, hepeviruses, hepadnaviruses, flaviviruses, parvoviruses, and picornaviruses. Using PCR methods, we first detected hepatovirus sequences in *Hipposideros larvatus* (3.85%). We also reported the first detection of Zika virus-related sequences in rats and house shrews. Sequences related to influenza A virus and herpesviruses were detected in liver. Higher detection rates of pegivirus sequences were found in liver tissue and serum samples from rats (7.85% and 15.79%, respectively) than from house shrews. Torque teno virus sequences had higher detection rates in the serum samples of rats and house shrews (52.72% and 5.26%, respectively) than in the liver. Near-full length genomes of pegivirus and torque teno virus were amplified. This study is the first to compare the viral communities in the liver of bats, rats, mice, and house shrews. Its findings expand our understanding of the virome in the liver of these animals and provide an insight into hepatitis-related viruses.

## Introduction

Viral hepatitis is a major global health concern. In 2015, 1.34 million persons died from the consequences of viral hepatitis. Hepatitis A-E viruses are the common causative agents of viral hepatitis and account for most of the cases of viral hepatitis (Yano et al., 2010). HAV and HEV are mainly transmitted through fecal–oral route (Augustine et al., 2020), and they both possess a wide range of natural hosts. Currently, the zoonotic potential of HAV is still unclear (Sander et al., 2018), while some hepatitis cases reported to date being linked to rat HEV (Sridhar et al., 2018; Andonov et al., 2019). Hepatitis B–D viruses are blood-borne viruses that cause liver diseases, including chronic hepatitis, cirrhosis, and hepatocellular carcinoma (Tu et al., 2017). In addition, some other viruses can also cause hepatitis, such as HPeV (Bigelow et al., 2016), adenovirus (Lynch et al., 2011), herpesvirus (Suto et al., 1995), DENV (Bowman et al., 2012), CHIKV (Bonifay et al., 2018) and ZIKV (Wu et al., 2017). Human liver diseases related to infection by the influenza A virus has been documented. In addition, a previous study showed that infection with the influenza A virus can cause hepatitis in an animal model (Morocco et al., 2014; Nonaka et al., 2019; Zhang et al., 2019). Some viruses, like pegivirus (Mrzljak and Tabain, 2019), TTV (Paraná and Lyra, 2000), KIs-V (Lole et al., 2017), and SEN-V (Sehgal, 2003), are considered to be the potential causes of hepatitis. However, the etiology of the remaining 10–20% hepatitis cases remains unclear.

Among the pathogens that cause human diseases, about 61% come from animals (Taylor et al., 2001), with viral infections accounting for two-thirds of the burden of infectious diseases (Nii-Trebi, 2017). Bats are reservoirs for many viruses and have served as the origin of several emerging viral infectious diseases, including SARS and MERS (Bolles et al., 2011; Lu et al., 2015). In addition, evidence indicates that the SARS-CoV-2, which causes COVID-19, might also come from bats (Zhou et al., 2020). Like bats, rats are also crucial to transmitting many human pathogenic viruses, such as HEV, hantaviruses and arenaviruses (Andonov et al., 2019; Gravinatti et al., 2020). Shrews are small mammals and can also spread many viruses to humans, with some old species commonly found near human residences, such as the *Suncus murinus* (Asian house shrew) (Li et al., 2016). Viruses related to the etiologies and potential etiologies of human hepatitis have been detected in the liver of these animals, including adenovirus, hepadnavirus, and hepevirus in bats; hepevirus in rats; and hepacivirus in house shrews (Wang et al., 2017; He et al., 2018; Jansen van Vuren and Allam, 2018; Guo et al., 2019; Maas et al., 2019). Influenza A virus was found in lung samples from wild rats (Cummings et al., 2019). However, none of the studies has investigated influenza A virus in liver tissue samples from rats and other animals. Therefore, understanding the viruses in the liver of bats, rats, and shrews is very important for the control and prevention of emerging zoonotic viruses and can provide an insight into the hepatitis-related viruses. However, to date, there has not been any report on viral communities in liver tissue samples from urban rats and house shrews or a comparison of the viral community composition of liver tissue samples among laboratory rats, mice, urban rats, house shrews, and bats.

In this study, we used the viral metagenomic technique to investigate and compare the viral community composition of liver tissue samples among laboratory rats, mice, urban rats, house shrews, and bats trapped in southern China. In addition, we surveyed the prevalence and genetic diversity of a set of viruses that associated with potential etiologies of hepatitis in these animals by using the PCR method.

## Materials and Methods

### Animal Capture and Sample Collection

**FIGURE 1:**
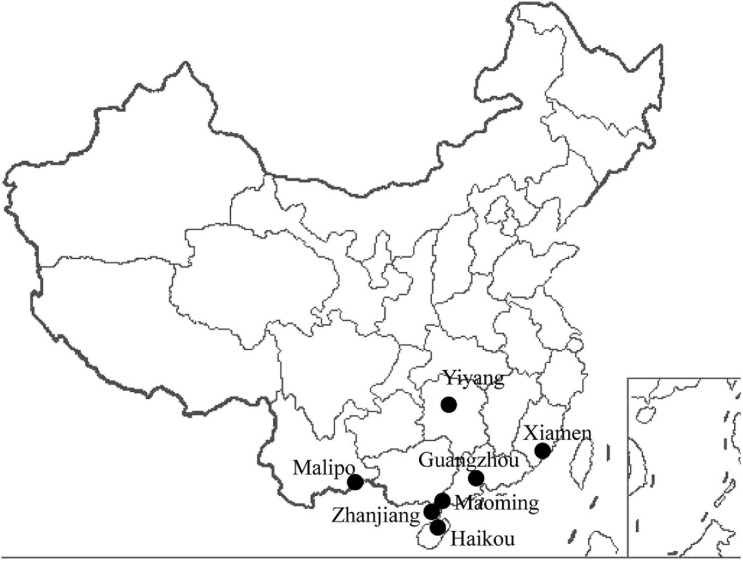
Locations of the animal trapping sites in China. Map source: https://image.so.com/view?q=%E4%B8%AD%E5%9B%BD%E5%9C%B0%E5%9B%BE&src=srp&correct=%E4%B8%AD%E5%9B%BD%E5%9C%B0%E5%9B%BE&ancestor=list&cmsid=50712c46325496ee4c7fbfbded18f06e&cmras=0&cn=0&gn=0&kn=50&crn=0&bxn=20&fsn=130&cuben=0&pornn=0&manun=50&adstar=0&clw=233#id=812433445ba11b77cba553308beab1d9&currsn=0&ps=136&pc=136

A total of 1,003 wild animals (177 bats, 624 urban rats, and 202 house shrews) were trapped in southern China between 2014 and 2018 (Figure 1 and Table 1). Urban rats and house shrews were captured near human residences with cage traps. Bats were trapped using mist nets or hand nets in residential areas, city parks, abandoned houses, and caves. Laboratory animals (five adult BALB/C mice and five adult SD rats) were obtained from the Animal Experiment Center, Southern Medical University, Guangzhou, China. Animals were anesthetized by 3% diethyl ether inhalation, and the dosage was adjusted according to their heart rate, respiratory frequency, corneal reflection, and extremity muscle tension. Trained personnel wore filtering facepiece respirators, chemical safety goggles, anti-static uniforms, and chemical protective gloves to protect them from diethyl ether. Blood was drawn by cardiac puncture and centrifuged to obtain serum samples. Then, animals were sacrificed by cervical dislocation. Liver tissue samples were soaked in RNAlater (Invitrogen, Carlsbad, CA, United States). The following samples were collected from the trapped animals: 247 serum samples and 1,003 liver tissue samples. All of the samples were stored at −80°C prior to processing. The species of each animal was identified by sequencing the *cytB* gene (Arai et al., 2008).

**TABLE 1:** Animals trapped in different places.

**Species**		**Hainan province**	**Hunan province**	**Fujian province**	**Yunnan province**	**Guangdong province**			**Total**
		**Haikou City**	**Yiyang City**	**Xiamen City**	**Malipo County**	**Zhanjiang City**	**Guangzhou City**	**Maoming City**	
Bats	*Hipposideros larvatus*	52	–	–	–	–	–	–	**52**
	*Rhinolophus affinis*	1	–	–	–	–	–	–	**1**
	*Rhinolophus pusillus*	1	–	–	–	–	–	–	**1**
	*Scotophilus kuhlii*	–	–	–	–	59	–	62	**121**
	*Pipistrellus abramus*	–		–	–	–	6	–	**6**
	*Cynopterus sphinx*	–		–	–	–	21	–	**21**
	Subtotal	**54**	–	–	–	**59**	**27**	**62**	**202**
Rats	*Rattus norvegicus*	–	88	35	51	–	201	86	**461**
	*Rattus tanezumi*	–	19	24	1	–	13	7	**64**
	*Rattus losea*	–	–	98	–	–	–	–	**98**
	*Bandicota indica*	–	–	1	–	–	–	–	**1**
	Subtotal	–	**107**	**158**	**52**	–	**214**	**93**	**624**
Shrews	*Suncus murinus*	–	–	10	–	–	167	–	**177**
	Total	**54**	**107**	**168**	**52**	**59**	**408**	**155**	**1003**

*Bold values indicates the total and subtotal numbers of the animals.*

### Metagenomic Sequencing and Analysis

Liver tissue samples from two species of urban rats and house shrews from Guangzhou, and four species of bats trapped in three different cities were randomly selected. Original samples from the trapped animals were pooled into seven groups according to the grouping strategy (Supplementary Table 1). Liver tissue samples from laboratory animals were also pooled into two groups and used as the baseline for viral diversity. A total of nine pooled samples were obtained.

To investigate the tropism of the viruses, and the blood contamination in liver tissue samples during animal dissection, liver and serum samples from 24 *Rattus norvegicus* (Norway rats) were randomly selected and pooled four groups (Supplementary Table 1).

All pooled samples were homogenized, centrifuged, and filtered through 0.22-μm filters. Non-particle-protected nucleic acid was digested with DNases and RNase (New England Biolabs, Ipswich, MA, United States). Then viral nucleic acids were extracted using MiniBEST Viral RNA/DNA Extraction Kits (TaKaRa Biotechnology, Kusatsu, Japan). Reverse transcription was performed with Transcriptor First Strand cDNA Synthesis Kits (Roche, Basel, Switzerland), using the primer described in a previous study (Allander et al., 2005), before obtaining the random PCR products (Allander et al., 2005). Libraries were constructed using the TruSeq DNA Sample Prep Kit (Illumina, San Diego, CA), and sequenced on an Illumina HiSeq at Shanghai Majorbio Bio-Pharm Technology (Shanghai, China) with 300 bp (2 × 150 bp) paired-end reads.

The quality score cut-off value was 20, sequences with ambiguous bases (more than 10 bp N) and short length reads (less than 50 bp) were removed by using Sickle^1^. To remove host-related sequences, quality reads were aligned with the host genome via BWA (Naccache et al., 2014). Reads with a high degree of similarity with the hosts’ genome were removed in further analyses. Then, reads (average length, 150 bp) were aligned with the sequences in the NCBI nucleotide sequence (NCBI NT) database^2^ by using BLASTN. In addition, short reads were assembled using IDBA-UD algorithm based on the de Bruijn graph approach (Peng et al., 2012). Assembled contigs were then filtered to a minimum length of 264 bp followed by gene prediction using Metagene (Noguchi et al., 2006). Protein-coding genes with a minimum length of 100 bp were retained. Then, genes were clustered to generate a non-redundant gene catalog using CD-HIT (95% identity and 90% overlap) (Fu et al., 2012). The resultant representative gene sequence clusters were subsequently taxonomically annotated based on the NCBI non-redundant protein sequence (NCBI NR) database^3^ by using BLASTP with a cut off value (E-value) of 1 × 10^–5^ (Altschul et al., 1997.; Xie et al., 2012; Yu and Zhang, 2013). To control laboratory-component-linked viral sequence contamination, the potential virus-related sequences were analyzed based on the results of a previous study (Asplund et al., 2019). Sequences that might link to laboratory-component viruses were removed (referred to Supplementary Tables 2–5 in the previous study) (Asplund et al., 2019). In addition, to mitigate the cross-library contamination due to index-hopping, the viral sequence was presumed to be a contaminant from another library and removed if the read count representing the abundance of the viral sequence was less than 0.1% of that representing the highest count for that viral sequence among the other libraries (Mahar et al., 2020; Pettersson and Ellström, 2020). Then, potential viral sequences related to retroviruses were analyzed again using BLAST, and the sequences with short length (less than 200 bp; <60% identity at the nucleotide level) were removed. The remaining sequences were classified into sequences related to vertebrate viruses, invertebrate viruses, plant viruses, phages, and others based on a healthy dose of common sense (Holmes, 2019). Since the animals included in our study are all mammals, we mainly analyzed the potential viral sequences related to mammalian viruses. Relative abundance was calculated by dividing the reads number mapped to genes to the total number of reads that could be annotated to known viruses in each sample (Shi et al., 2015; Mishra et al., 2019; Hameed et al., 2020). The relative abundance of the sequences related to a taxonomic group was summed up by the abundance of its matching genes. Alpha diversity indexes were analyzed using R software at the genus level, including Chao, abundance-based coverage estimators (ACE), Shannon, and Simpson parameters. Beta diversity was analyzed to investigate the similarity of the viral community composition among the samples (genus level) and visualized via principal component analysis (PCA). PCA was performed based on Euclidean distance by using R software (vegan package). Phylogenetic analyses were performed with Molecular Evolutionary Genetic Analysis (MEGA) software version 6.0 (Oxford Molecular Ltd., Cambridge, United Kingdom) using the maximum likelihood method.

### Detection and Sequencing of Viruses Associated With Several Etiologies and Potential Etiologies of Human Hepatitis

Nucleic acids from original samples were extracted using MiniBEST Viral RNA/DNA Extraction Kits. Hepatovirus, hepacivirus, hepatitis D virus (HDV), parechovirus, herpesvirus, influenza A virus, adenovirus, DENV, CHIKV, ZIKV, pegivirus, KIs-V, TTV, and SEN-V were detected by using PCR methods (VanDevanter et al., 1996; Wellehan et al., 2004; Moureau et al., 2007; Harvala et al., 2008; Satoh et al., 2011; Quan et al., 2013; Firth et al., 2014; Pirouzi et al., 2014; Drexler et al., 2015; Lee et al., 2016; Huo et al., 2017; Abundes-Gallegos et al., 2018; Binh et al., 2018; Xiong et al., 2018). The LV was detected using an in-house method (Supplementary Table 2).

Large fragment sequences and full-length genomes of these viruses were amplified (Supplementary Table 2) (Xiong et al., 2018, 2019).

All of the amplified products were separated on a 1.5% agarose gel and the positive samples were sent to the Beijing Genomics Institute for sequencing.

### Detection of the Replication of Pegivirus

To survey the replication of pegivirus in the liver, minus-strand pegivirus RNA was detected in serum and liver tissue samples from the pegivirus positive animals by using the method reported in a previous study (Tucker et al., 2000).

### Phylogenetic Analysis

Sequences obtained in our study were selected and aligned with the related sequences obtained on GenBank using MAFFT (Katoh et al., 2002). Phylogenetic trees were constructed using MrBayes software (version 3.2^4^) based on GTR + G + I nucleotide substitution matrix (Ronquist et al., 2012; Abadi et al., 2019). We set the Ngen in our study as 2,000,000 with sampling every 100 generations. A 25% burn-in value was set for the final trees. The convergence was assessed by the average standard deviation of split frequencies (ASDSF) and potential scale reduction factor (PSRF). As runs converged, ASDSF and PSRF approached 0.0 and 1.0, respectively (Ronquist et al., 2012).

### Statistical Analysis

Statistical analysis was performed using the Statistical Product and Service Solutions software (SPSS, version 13.0; IBM Corp., Armonk, NY, United States). Descriptive statistics (Crosstabs) were calculated to assess viral prevalence. Chi-square tests were performed to test differences in viral prevalence between the animals. *P* < 0.05 was considered to be statistically significant.

### Ethical Guidelines

The study protocol was approved by the Animal Ethics and Welfare Committee of the School of Public Health, Southern Medical University and adhered to the guidelines for the Rules for the Implementation of Laboratory Animal Medicine (1998) from the Ministry of Health, China. All surgical procedures were performed under anesthesia to minimize suffering. Endangered or protected species were not included in this study.

## Results

### Viral Communities in the Liver of Different Animals

**FIGURE 2:**
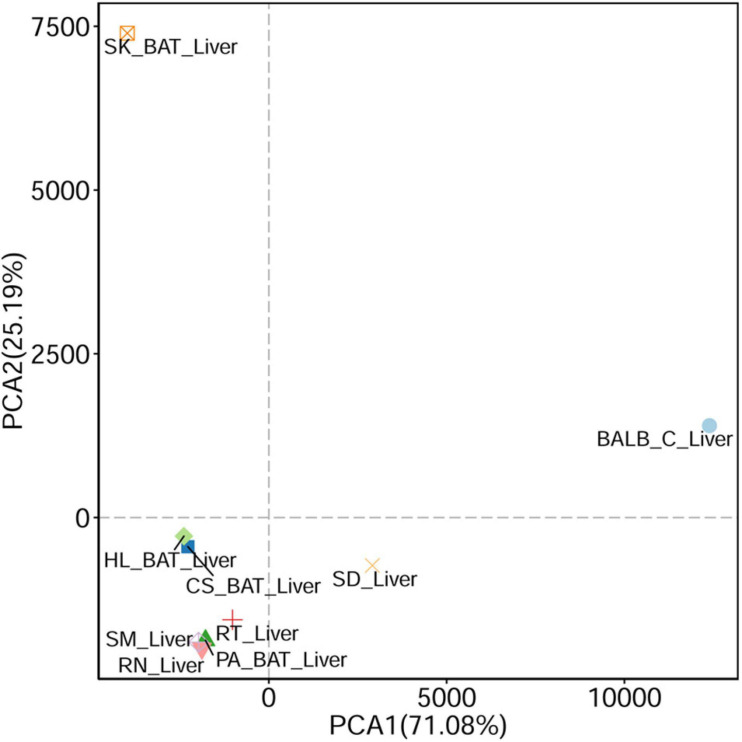
Principal component analysis (PCA) (based on Euclidean distance) plots between liver tissue samples from different animals.

Based on the PCA analysis, viral communities within the urban rats were similar (Figure 2). Compared with laboratory animals, a higher similarity was found in viral compositions between urban rats and SD rats than between urban rats and BALB/C mice (Figure 2). We also found a high similarity in viral composition between urban rats and house shrews. Among bats, the viral composition of *Cynopterus sphinx* (short-nosed fruit bats) was similar to that of *Hipposideros larvatus* (intermediate round leaf bats). Interestingly, the viral composition of *Pipistrellus abramus* (Japanese house bats) showed high similarity with urban rats and house shrews. *Scotophilus kuhlii* (Lesser Asiatic yellow bats) showed large differences in their viral composition from other animals (Figure 2).

Large number of reads were obtained in our study (Supplementary Table 3). The number of reads that elicited no results in bats, urban rats, and house shrews accounted for 85.24%, 86.67%, and 74.27%, respectively, of the total number of reads. Potential viral sequences that might be associated with laboratory-component-linked contamination were removed, accounting for 21.88%, 5.78%, and 5.22% of the sequences related to known viruses in house shrews, urban rats and bats, respectively (Asplund et al., 2019). After filtered the viral sequences, the remaining sequences detected in the liver samples were still related to a large number of viruses, including vertebrate viruses, invertebrate viruses, plant viruses, phages, and others (Supplementary Figure 1). Sequences from urban rats were related to more mammalian viruses than any other species of animals (Supplementary Tables 4, 5). Mammalian virus-related sequences detected in urban rats were related to 17 viral families and 26 viral genera. In laboratory animals, we only found sequences related to 15 viral families and 18 viral genera. Sequences obtained from house shrews were related to 12 viral families and 18 viral genera. In bats, the detected sequences were related to 14 viral families and 24 viral genera. Generally, sequences related to retroviruses and herpesviruses were common in animals (Supplementary Figure 2). A high relative abundance of the sequences related to orthomyxoviruses was found in urban rats. Higher relative abundance of sequences related to alphainfluenzaviruses (100% identity at the nucleotide level) was detected in urban rats at the genus level, while sequences related to betaretroviruses were common in house shrews, and a higher relative abundance of the sequences related to roseoloviruses was found in bats (Supplementary Figure 3). In addition, sequences related to percaviruses (75% identity at the nucleotide level) were only detected in bats. Sequences related to several hepatitis-associated viruses were detected, including herpesviruses (66% identity at the nucleotide level), influenza A virus (100% identity at the nucleotide level), and HEV (78% identity at the nucleotide level). Viral sequences related to the potential causes of hepatitis were also found, such as TTV (89% identity at the nucleotide level) and pegivirus (93% identity at the nucleotide level).

### Viral Communities Between the Liver Tissue and Serum Samples From *R. norvegicus*

**FIGURE 3:**
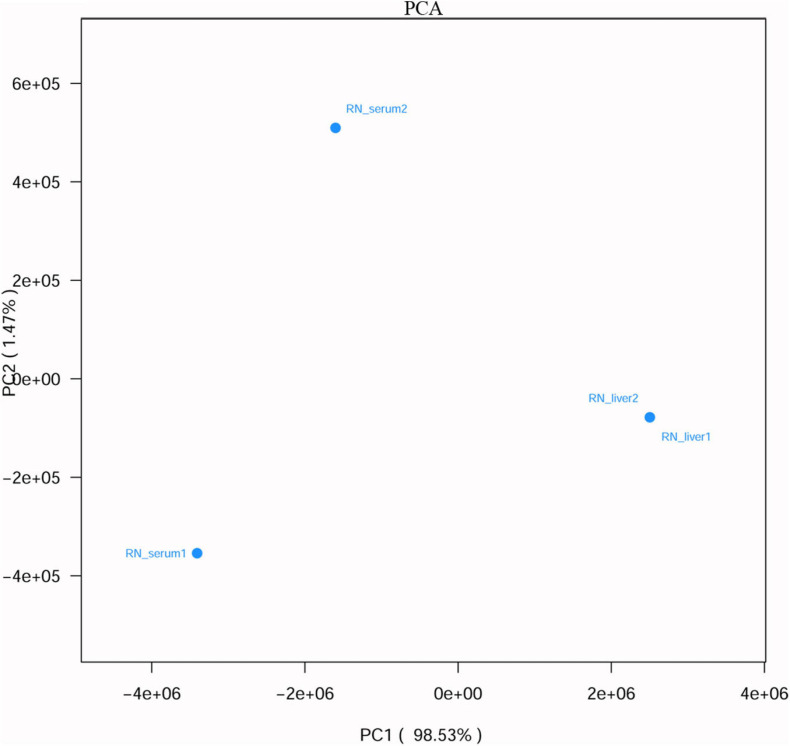
Principal component analysis (PCA) (based on Euclidean distance) plots between liver tissue and serum samples from *R. norvegicus*.

To figure out whether the viruses have liver tropism or not, virome in the serum and liver tissue samples from *R. norvegicus* were revealed and compared. Viral communities in the blood samples showed a high difference from liver tissue samples (Figure 3 and Supplementary Table 6).

Higher relative abundance of the sequences related to anelloviruses and flaviviruses were found in serum samples than liver tissue samples, while liver tissue samples had higher relative abundance of the sequences related to retroviruses and herpesviruses (Supplementary Figure 4). We found a higher relative abundance of sequences related to pegiviruses in serum samples at the genus level. Still, sequences related to roseoloviruses, gammaretroviruses and betaretroviruses were more common in liver tissue samples (Supplementary Figure 5). It seems that some animal viruses associated with the etiologies and potential etiologies of human hepatitis, such as herpesviruses, did have a liver tropism. Interestingly, sequences related to rhinovirus C (94% identity at the nucleotide level), which is not considered to be associated with hepatitis, were only detected in the liver tissue samples (Supplementary Table 7). However, higher relative abundance of the sequences related to anelloviruses (89% identity at the nucleotide level, Supplementary Figure 4) and pegiviruses (93% identity at the nucleotide level, Supplementary Figure 5) were found in serum samples. In addition, sequences related to hepacivirus from *Bradypus variegatus* (76% identity at the nucleotide level) were only found in serum samples (Supplementary Table 7 and Supplementary Figure 6).

### PCR Screening

Since a large number of virus-related sequences had been detected in the liver tissue samples, and some of these sequences were related to the etiologies and potential etiologies of hepatitis; confirmatory PCR tests were used to investigate prevalence and genetic diversity of several animal viruses related to the etiologies and potential etiologies of hepatitis, including hepatovirus, hepacivirus, hepatitis D virus (HDV), influenza A virus, adenovirus, herpesvirus, parechovirus, DENV, ZIKV, CHIKV, pegivirus, TTV, LV, KIs-V, and SEN-V (Supplementary Table 8).

In bats, we only found sequences related to hepatovirus (0.99%), influenza A virus (2.97%), and herpesvirus (20.79%); however, in urban rats and house shrews, we amplified the sequences related to a larger number of viruses, including adenovirus, ZIKV, influenza A virus, herpesviruses, pegivirus and TTV (Table 2). Sequences related to adenovirus were detected in one of the liver tissue samples from rats, and sequences related to ZIKV were only detected in urban rats (0.64%) and house shrews (1.13%). This is the first detection of the sequences related to ZIKV in urban rats and house shrews to the best of our knowledge. Sequences related to influenza A virus and herpesviruses were only detected in the liver tissue samples. The positive rate of sequences related to influenza A virus was significantly higher in house shrews than that in urban rats (43.50% vs. 2.88%, χ^2^ = 217.844, *P* < 0.05), while sequences related to herpesviruses had a significantly higher detection proportion in urban rats than house shrews (21.96% vs. 2.26%, χ^2^ = 57.841, *P* < 0.05). None of the samples was positive in detecting sequences related to hepacivirus, HDV, parechovirus, CHIKV, and DENV (Table 2).

**TABLE 2:** Detection of the viral sequences in animals.

		**Bats**							**Rats**					**Shrews**	
		***Hipposideros larvatus***	***Rhinolophus affinis***	***Rhinolophus pusillus***	***Scotophilus kuhlii***	***Pipistrellus abramus***	***Cynopterus sphinx***	**Subtotal**	***Rattus norvegicus***	***Rattus tanezumi***	***Rattus losea***	***Bandicota indica***	**Subtotal**	***Suncus murinus***	**Total**
Hepatovirus	Liver tissue sample	3.85 (2/52)	0 (0/1)	0 (0/1)	0 (0/121)	0 (0/6)	0 (0/21)	**0.99 (2/202)**	0 (0/461)	0 (0/64)	0 (0/98)	0 (0/1)	**0 (0/624)**	0 (0/177)	**0.20 (2/1003)**
	Serum sample	–	–	–	–	–	–	–	0 (0/170)	0 (0/20)	0 (0/38)	–	**0 (0/228)**	0 (0/19)	**0 (0/247)**
Hepacivirus	Liver tissue sample	0 (0/52)	0 (0/1)	0 (0/1)	0 (0/121)	0 (0/6)	0 (0/21)	**0 (0/202)**	0 (0/461)	0 (0/64)	0 (0/98)	0 (0/1)	**0 (0/624)**	0 (0/177)	**0 (0/1003)**
	Serum sample	–	–	–	–	–	–	–	0 (0/170)	0 (0/20)	0 (0/38)	–	**0 (0/228)**	0 (0/19)	**0 (0/247)**
Hepatitis D virus	Liver tissue sample	0 (0/52)	0 (0/1)	0 (0/1)	0 (0/121)	0 (0/6)	0 (0/21)	**0 (0/202)**	0 (0/461)	0 (0/64)	0 (0/98)	0 (0/1)	**0 (0/624)**	0 (0/177)	**0 (0/1003)**
	Serum sample	–	–	–	–	–	–	–	0 (0/170)	0 (0/20)	0 (0/38)	–	**0 (0/228)**	0 (0/19)	**0 (0/247)**
Adenovirus	Liver tissue sample	0 (0/52)	0 (0/1)	0 (0/1)	0 (0/121)	0 (0/6)	0 (0/21)	**0 (0/202)**	0(0/461)	0 (0/64)	1.02 (1/98)	0 (0/1)	**0.16 (1/624)**	0 (0/177)	**0.10 (1/1003)**
	Serum sample	–	–	–	–	–	–	–	0 (0/170)	0 (0/20)	0 (0/38)	–	**0 (0/228)**	0 (0/19)	**0 (0/247)**
Influenza A virus	Liver tissue sample	5.77 (3/52)	0 (0/1)	100 (1/1)	1.65 (2/121)	0 (0/6)	0 (0/21)	**2.97 (6/202)**	3.47 (16/461)	1.56 (1/64)	1.02 (1/98)	0 (0/1)	**2.88 (18/624)**	43.50 (77/177)	**10.06 (101/1003)**
	Serum sample	–	–	–	–	–	–	–	0 (0/170)	0 (0/20)	0 (0/38)	–	**0 (0/228)**	0 (0/19)	**0 (0/247)**
Herpesvirus	Liver tissue sample	15.38 (8/52)	0 (0/1)	0 (0/1)	28.10 (34/121)	0 (0/6)	0 (0/21)	**20.79 (42/202)**	24.30 (112/461)	17.19 (11/64)	14.28 (14/98)	0 (0/1)	**21.96 (137/624)**	2.26 (4/177)	**18.24 (183/1003)**
	Serum sample	–	–	–	–	–	–	–	0 (0/170)	0 (0/20)	0 (0/38)	–	**0 (0/228)**	0 (0/19)	**0 (0/247)**
Parechovirus	Liver tissue sample	0 (0/52)	0 (0/1)	0 (0/1)	0 (0/121)	0 (0/6)	0 (0/21)	**0 (0/202)**	0 (0/461)	0 (0/64)	0 (0/98)	0 (0/1)	**0 (0/624)**	0 (0/177)	**0 (0/1003)**
	Serum sample	–	–	–	–	–	–	–	0 (0/170)	0 (0/20)	0 (0/38)	–	**0 (0/228)**	0 (0/19)	**0 (0/247)**
Chikungunya virus	Liver tissue sample	0 (0/52)	0 (0/1)	0 (0/1)	0 (0/121)	0 (0/6)	0 (0/21)	**0 (0/202)**	0 (0/461)	0 (0/64)	0 (0/98)	0 (0/1)	**0 (0/624)**	0 (0/177)	**0 (0/1003)**
	Serum sample	–	–	–	–	–	–	–	0 (0/170)	0 (0/20)	0 (0/38)	–	**0 (0/228)**	0 (0/19)	**0 (0/247)**
Dengue virus	Liver tissue sample	0 (0/52)	0 (0/1)	0 (0/1)	0 (0/121)	0 (0/6)	0 (0/21)	**0 (0/202)**	0 (0/461)	0 (0/64)	0 (0/98)	0 (0/1)	**0 (0/624)**	0 (0/177)	**0 (0/1003)**
	Serum sample	–	–	–	–	–	–	–	0 (0/170)	0 (0/20)	0 (0/38)	–	**0 (0/228)**	0 (0/19)	**0 (0/247)**
Zika virus	Liver tissue sample	0 (0/52)	0 (0/1)	0 (0/1)	0 (0/121)	0 (0/6)	0 (0/21)	**0 (0/202)**	0.87 (4/461)	0 (0/64)	0 (0/98)	0 (0/1)	**0.64 (4/624)**	1.13 (2/177)	**0.60 (6/1003)**
	Serum sample	–	–	–	–	–	–	–	0 (0/170)	0 (0/20)	0 (0/38)	–	**0 (0/228)**	0 (0/19)	**0 (0/247)**
Ljungan virus	Liver tissue sample	0 (0/52)	0 (0/1)	0 (0/1)	0 (0/121)	0 (0/6)	0 (0/21)	**0 (0/202)**	0 (0/461)	0 (0/64)	0 (0/98)	0 (0/1)	**0 (0/624)**	0 (0/177)	**0 (0/1003)**
	Serum sample	–	–	–	–	–	–	–	0 (0/170)	0 (0/20)	0 (0/38)	–	**0 (0/228)**	0 (0/19)	**0 (0/247)**
Pegivirus	Liver tissue sample	0 (0/52)	0 (0/1)	0 (0/1)	0 (0/121)	0 (0/6)	0 (0/21)	**0 (0/202)**	9.11 (42/461)	3.12 (2/64)	5.10 (5/98)	0 (0/1)	**7.85 (49/624)**	5.65 (10/177)	**5.88 (59/1003)**
	Serum sample	–	–	–	–	–	–	–	18.24 (31/170)	15.00 (3/20)	5.26 (2/38)	–	**15.79 (36/228)**	5.26 (1/19)	**14.98 (37/247)**
Torque teno virus	Liver tissue sample	0 (0/52)	0 (0/1)	0 (0/1)	0 (0/121)	0 (0/6)	0 (0/21)	**0 (0/202)**	60.09 (277/461)	39.06 (25/64)	17.35 (17/98)	0 (0/1)	**51.12 (319/624)**	0.56 (1/177)	**31.90 (320/1003)**
	Serum sample	–	–	–	–	–	–	–	60.00 (102/170)	40.00 (8/20)	42.10 (16/38)	–	**52.72 (126/239)**	5.26 (1/19)	**51.42 (127/247)**
SEN-virus	Liver tissue sample	0 (0/52)	0 (0/1)	0 (0/1)	0 (0/121)	0 (0/6)	0 (0/21)	**0 (0/202)**	0 (0/461)	0 (0/64)	0 (0/98)	0 (0/1)	**0 (0/624)**	0 (0/177)	**0 (0/1003)**
	Serum sample	–	–	–	–	–	–	–	0 (0/170)	0 (0/20)	0 (0/38)	–	**0 (0/228)**	0 (0/19)	**0 (0/247)**
KIs-virus	Liver tissue sample	0 (0/52)	0 (0/1)	0 (0/1)	0 (0/121)	0 (0/6)	0 (0/21)	**0 (0/202)**	0 (0/461)	0 (0/64)	0 (0/98)	0 (0/1)	**0 (0/624)**	0 (0/177)	**0 (0/1003)**
	Serum sample	–	–	–	–	–	–	–	0 (0/170)	0 (0/20)	0 (0/38)	–	**0 (0/228)**	0 (0/19)	**0 (0/247)**
															

*Bold values indicates the total and subtotal numbers of the animals.*

When detecting the animal viruses related to the potential causes of human hepatitis, we found sequences related to pegivirus and TTV in both the serum and liver tissue samples from urban rats and house shrews. Detection rates of sequences related to pegivirus and TTV were higher in serum samples (14.98% and 51.42%) than in liver tissue samples (5.88% and 31.90%). Higher detection rates of sequences related to pegivirus and TTV were measured in livers from urban rats (7.85% and 51.12%) compared with those from house shrews (5.65% and 0.56%). Interestingly, combining with the data from our previous study (He et al., 2018), a significantly higher detection rate of TTV sequences was found in HEV-positive animals than in HEV-negative animals (50.40% vs. 38.02%, χ^2^ = 6.742, *P* < 0.05), however, we did not find the similar result in pegivirus sequences. To figure out the replication of pegivirus in the liver, liver and serum samples from 79 pegivirus positive animals were used to detect minus-strand pegivirus RNA. Of 79 pegivirus positive animals, only 25 serum samples were enough for viral DNA/RNA extraction (more than 200 μl). Minus-strand pegivirus RNA was only found in *R. norvegicus* (Table 3). Thirteen of them found minus-strand pegivirus RNA in both liver and serum samples, while six Norway rats only had minus-strand pegivirus RNA in serum samples. None of the samples was positive in detecting sequences related to the other three potential causes (LV, KIs-V, SEN-V) of human hepatitis.

**TABLE 3:** Minus-strand pegivirus RNA in pegivirus positive animals.

	**XM10**	**XM16**	**XM50**	**XM111**	**GZ66**	**GZ224**	**GZ260**	**GZ324**	**GZ325**	**GZ326**	**GZ331**	**GZ333**	**GZ434**	**GZ435**	**GZ461**	**GZ462**	**GZ470**	**GZ486**	**MM61**	**MM87**	**YN18**	**YN33**	**YN34**
Liver	N	Y	Y	Y	Y	Y	Y	Y	Y	Y	Y	Y	N	Y	Y	Y	N	N	N	Y	Y	Y	N
Serum	Y	Y	–	Y	–	Y	Y	Y	Y	Y	–	Y	Y	Y	Y	Y	Y	Y	Y	Y	–	Y	Y

*Y, Presence of the minus-strand pegivirus RNA.*

*N, Absence of the minus-strand pegivirus RNA.*

*–, Serum samples that are not enough for nucleic acid extraction.*

### Phylogenetic Analysis

**FIGURE 4:**
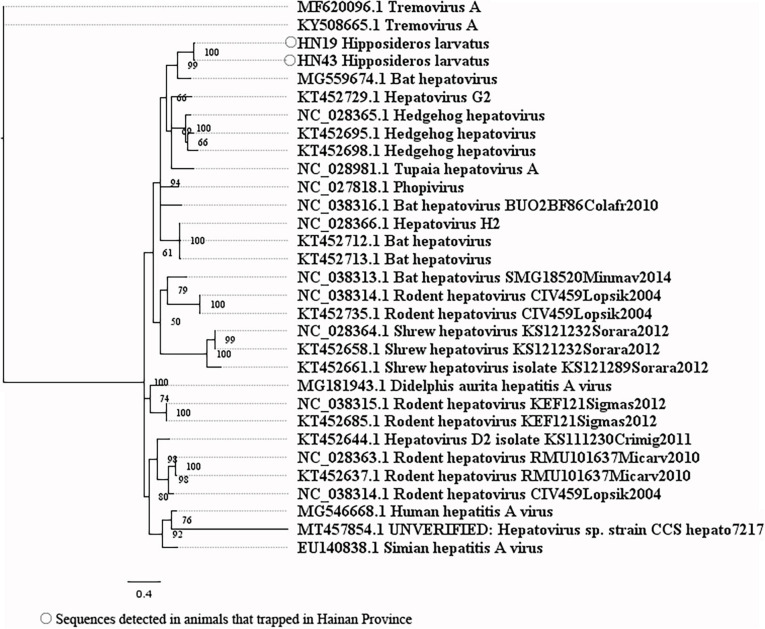
Phylogenetic tree constructed based on PCR-screened nucleotide sequences (333 bp) of hepatovirus from bats (MrBayes, GTR + G + I nucleotide substitution model). A total of 27 representative hepatovirus sequences (polyprotein gene region) belonging to different species within genus *Hepatovirus* are included for comparison. Two tremovirus sequences are set as outgroup. Percentages of the posterior probability (PP) values are indicated.

**FIGURE 5:**
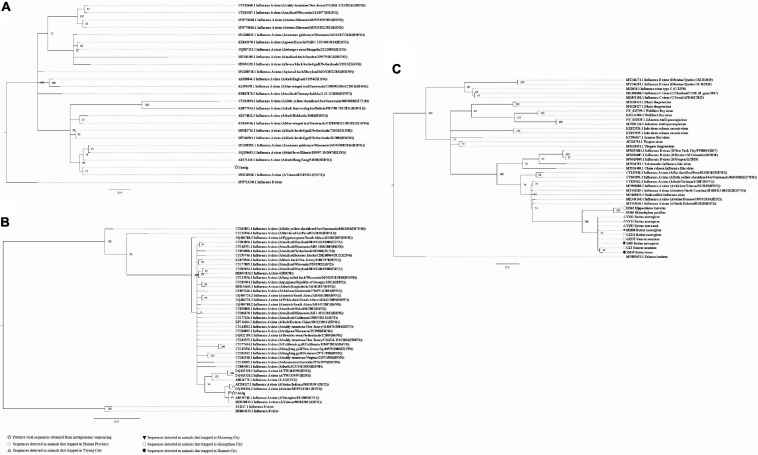
**(A)** Phylogenetic tree constructed based on potential viral nucleotide sequence (148 bp, neuraminidase gene) related to influenza A virus that obtained by using metagenomic sequencing (MrBayes, GTR + G + I nucleotide substitution model). A total of 23 representative sequences belonging to different species within genus *Alphainfluenzavirus* are included for comparison, one sequence belonging to genus *Betainfluenzavirus* is set as outgroup. Percentages of the posterior probability (PP) values are indicated. **(B)** Phylogenetic tree constructed based on potential viral nucleotide sequence (150 bp, nucleocapsid protein gene) related to influenza A virus that obtained by using metagenomic sequencing (MrBayes, GTR + G + I nucleotide substitution model). A total of 39 representative sequences belonging to different species within genus *Alphainfluenzavirus* are included for comparison, two sequences belonging to genus *Betainfluenzavirus* are set as outgroup. Percentages of the posterior probability (PP) values are indicated. **(C)** Phylogenetic tree constructed based on PCR-screened nucleotide sequences (156 bp) of influenza A virus from urban rats, bats, and house shrews (MrBayes, GTR + G + I nucleotide substitution model). A total of 30 representative sequences (matrix region) belonging to different genera within family Orthomyxoviridae are included for comparison. One sequence of salmon isavirus is set as outgroup. Percentages of the posterior probability (PP) values are indicated.

**FIGURE 6:**
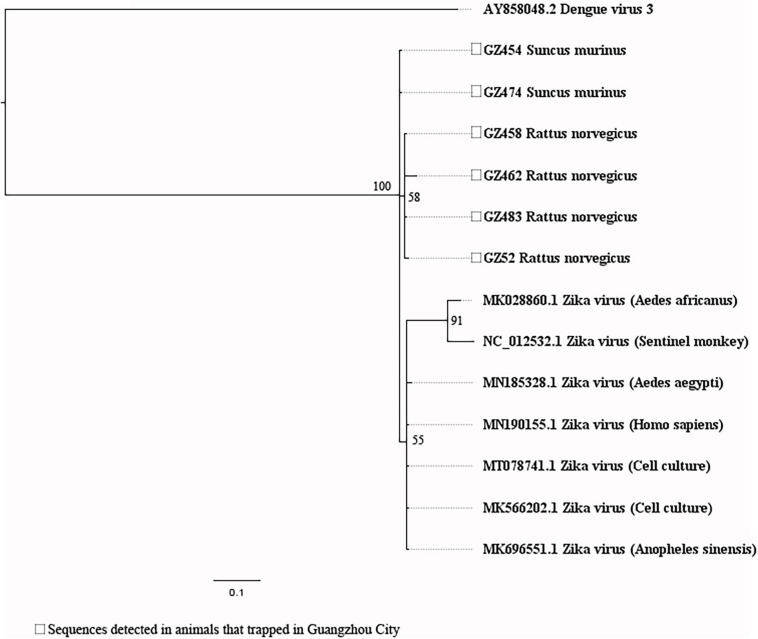
Phylogenetic tree constructed based on PCR-screened nucleotide sequences (156 bp) of Zika virus (ZIKV) from urban rats and house shrews (MrBayes, GTR + G + I nucleotide substitution model). A total of seven representative sequences of ZIKV derived from human, *Aedes aegypti*, and *Anopheles sinensis* are included for comparison (polyprotein gene region). One dengue virus sequence is set as outgroup (polyprotein gene region). Percentages of the posterior probability (PP) values are indicated.

**FIGURE 7:**
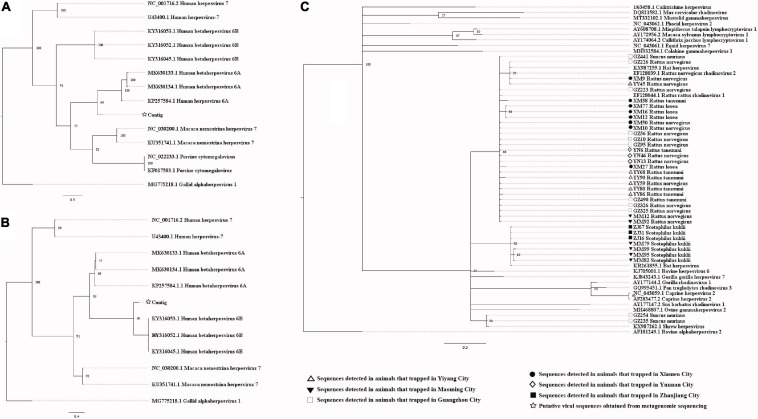
**(A)** Phylogenetic tree constructed based on potential viral nucleotide sequence (451 bp, IE-2 protein gene) related to human herpesvirus 6A that obtained by using metagenomic sequencing (MrBayes, GTR + G + I nucleotide substitution model). A total of 10 representative sequences belonging to different species within genus *Roseolovirus* are included for comparison, and one sequences belonging to genus *Iltovirus* is set as outgroup. Percentages of the posterior probability (PP) values are indicated. **(B)** Phylogenetic tree constructed based on potential viral nucleotide sequence (245 bp) related to human herpesvirus 6B that obtained by using metagenomic sequencing (MrBayes, GTR + G + I nucleotide substitution model). A total of 12 representative sequences belonging to different species within genus *Roseolovirus* are included for comparison, and one sequences belonging to genus *Iltovirus* is set as outgroup. Percentages of the posterior probability (PP) values are indicated. **(C)** Phylogenetic tree constructed based on PCR-screened nucleotide sequences (144 bp) of herpesviruses from urban rats, bats, and house shrews (MrBayes, GTR + G + I nucleotide substitution model). A total of 23 representative sequences (DPOL gene region) are included for comparison, including 20 sequences belonging to different genera within subfamily Gammaherpesvirinae, one bat herpesvirus sequence, one shrew herpesvirus sequence, and one alphaherpesvirus sequence that is set as outgroup. Percentages of the posterior probability (PP) values are indicated.

**FIGURE 8:**
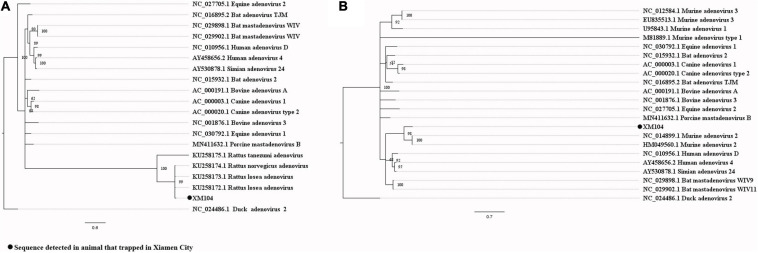
**(A)** Phylogenetic tree constructed based on PCR-screened nucleotide sequence (294 bp) of adenovirus from *R. losea* (MrBayes, GTR + G + I nucleotide substitution model). A total of 18 representative mastadenovirus sequences and one aviadenovirus sequence (set as outgroup) are included for comparison (DNA polymerase gene region). Percentages of the posterior probability (PP) values are indicated. **(B)** Phylogenetic tree constructed based on the large nucleotide sequence (690 bp) of adenovirus from *R. losea* (MrBayes, GTR + G + I nucleotide substitution model). A total of 20 representative adenovirus sequences belonging to different species within genus *Mastadenovirus* and one aviadenovirus sequence (set as outgroup) are included for comparison (DNA polymerase gene region). Percentages of the posterior probability (PP) values are indicated.

Our attempts to amplify the large genomic fragments of hepatovirus, influenza A virus, ZIKV and herpesvirus failed, so phylogenetic analyses of these viruses were performed based on the PCR-screened sequences and the potential virus-related sequences obtained from viral metagenomics. The PCR-screened sequences related to hepatovirus in bats were similar to a hepatovirus sequence from *Hipposideros armiger* (MG559674.1, 81.9% and 100% at the nucleotide and amino acid levels, respectively), and they were also clustered together (Figure 4). Sequences related to influenza A virus from viral metagenomics showed a high degree of similarity with the neuraminidase gene (MH328920.1, 148 bp in length, 100% identity at the nucleotide level, Figure 5A) and nucleocapsid protein gene (MH328833.1, 150 bp in length, 100% identity at the nucleotide level, Figure 5B) of the influenza A virus. However, a relatively low similarity (42% identity at the nucleotide level) was found between the PCR-screened sequences (average length, 164 bp) and the matrix gene sequences of the influenza A virus from GenBank (Supplementary Tables 9, 10). PCR-screened sequences from urban rats and house shrews were clustered together, while sequences from bats formed an independent cluster (Figure 5C). These sequences formed a clade with influenza A virus detected in other species of animals. PCR-screened sequences related to ZIKV (average length, 158 bp) were detected in different species of animals and clustered together. They were similar to the polyprotein gene of the ZIKV isolated from human, non-human primates, and mosquitoes (Figure 6 and Supplementary Table 11). Using metagenomic sequencing, potential viral sequences related to human herpesviruses (genus *Roseolovirus* within the subfamily Betaherpesvirinae) were detected (Figures 7A,B). However, using PCR method, we found viral sequences related to rat, bat, and shrew herpesviruses. PCR-screened sequences detected in the same species of animals clustered together usually (Figure 7C). Interestingly, we found one sequence from house shrews (GZ441) that clustered with rat rhadinovirus sequences, suggesting cross-species transmission potential. Sequences belonging to the genus *Rhadinovirus* within the subfamily Gammaherpesvirinae were detected in urban rats and one house shrew (GZ441). In addition, in liver tissue samples from bats and most house shrews, we also found sequences belonging to *Bat herpesvirus* within family Herpesviridae and *Shrew herpesvirus* within order Herpesvirales, respectively. The PCR-screened sequence of adenovirus from *Rattus losea* was clustered with rat adenovirus sequences (Figure 8A), longer genomic sequence (690 bp in length) of it was clustered with murine adenovirus 2 sequences, which belonged to *Murine mastadenovirus B* within genus *Mastadenovirus* (Figure 8B).

**FIGURE 9:**
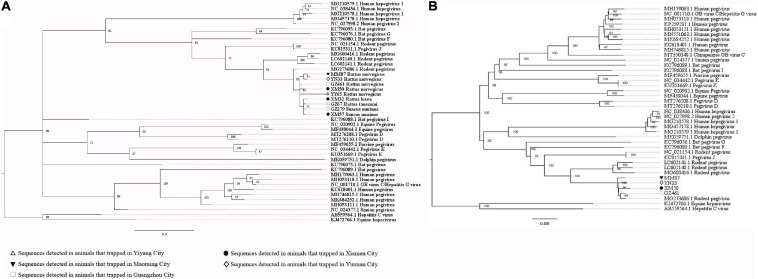
**(A)** Phylogenetic tree constructed based on PCR-screened nucleotide sequences (183 bp) of pegivirus from urban rats and house shrews (MrBayes, GTR + G + I nucleotide substitution model). A total of 33 representative pegivirus sequences of different species within genus *Pegivirus* and two sequences belonging to genus *Hepacivirus* (set as outgroup) are included for comparison (polyprotein gene region). Percentages of the posterior probability (PP) values are indicated. **(B)** Phylogenetic tree constructed based on near full-length nucleotide sequences (9912 bp) of pegivirus from *R. norvegicus* (MrBayes, GTR + G + I nucleotide substitution model). A total of 34 representative pegivirus sequences of different species within genus *Pegivirus* and two sequences belonging to genus *Hepacivirus* (set as outgroup) are included for comparison. Percentages of the posterior probability (PP) values are indicated.

**FIGURE 10:**
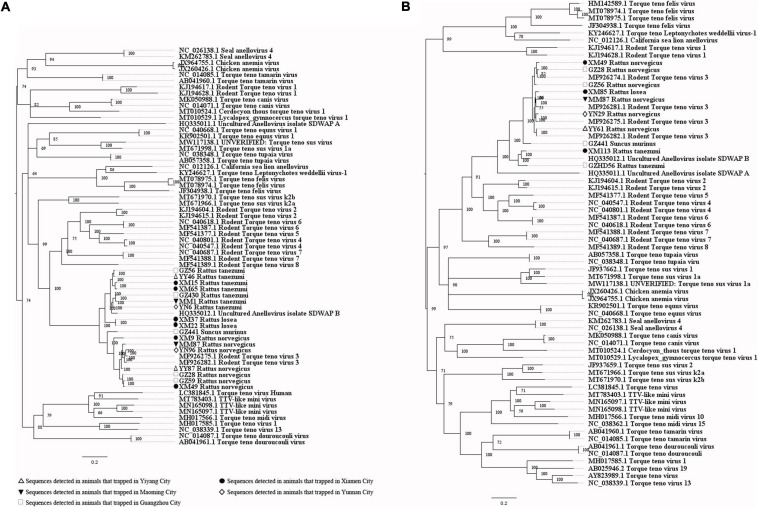
**(A)** Phylogenetic tree constructed based on PCR-screened nucleotide sequences (651 bp) of TTV from urban rats and house shrews (MrBayes, GTR + G + I nucleotide substitution model). A total of 48 representative sequences of different genera within family Anelloviridae are included for comparison (ORF1 region). Percentages of the posterior probability (PP) values are indicated. This tree is rooted using the model and not by the reference to an outgroup. **(B)** Phylogenetic tree constructed based on near full-length nucleotide sequences (2190 bp) of TTV from urban rats and house shrews (MrBayes, GTR + G + I nucleotide substitution model). A total of 56 representative sequences of different genera within family Anelloviridae are included for comparison. Percentages of the posterior probability (PP) values are indicated. This tree is rooted using the model and not by the reference to an outgroup.

When analyzing the sequences related to the potential causes of hepatitis, the PCR-screened sequences of pegivirus obtained from different species of animals were clustered together (Figure 9A). Four near full-length genomic sequences were obtained (GenBank accession numbers: MW055871-MW055874), with a high similarity between them (Supplementary Table 12). The near full-length genomic sequences of pegivirus both came from *R. norvegicus* and belonged to *Pegivirus J* (rodent pegivirus) (Figure 9B). The first near full-length TTV genomes from *Rattus tanezumi* (Asian house rats) and *R. losea*, and the first large genomic fragment (1757 bp) of TTV from *S. murinus* were obtained in this study. All of these sequences (GenBank accession numbers: MW055875-MW055884) belonged to rodent TTV genotype 3. TTV sequences detected in the same species of animals were usually clustered together. Still, we also found one sequence from the *Rattus losea* (XM85) that showed high similarity with the sequences from the *R. norvegicus*, which indicated the potential of cross-species transmission of this virus (Figure 10 and Supplementary Tables 13, 14).

## Discussion

To the best of our knowledge, this is the first study to reveal and compare viral communities in liver tissue samples from laboratory rats, mice, urban rats, house shrews, and bats using the viral metagenomic technique. The uppermost advantage of the metagenomic technique is its power to study many microorganisms in a single experiment which cannot be achieved by using conventional microbiology methods. However, it can be less accurate for defining microbial species/strains due to short read lengths (J. Zhang et al., 2020). In general, the species of viruses can be determined based on the long gene segments obtained by assembling short reads in viral metagenomics (Shi et al., 2016). Since strict standards for the length and identity of the sequences obtained from viral metagenomics were not set in our study, further confirmatory tests, such as PCR, were needed (Cantalupo and Pipas, 2019; Holmes, 2019). Because our research focused mainly on some viruses related to the etiologies and potential etiologies of human hepatitis, confirmatory tests using PCR were undertaken on only 15 animal viruses related to the causes or potential causes of human hepatitis. Not all the annotated viral species in viral metagenomics were verified by carrying out confirmatory PCR tests. Therefore, we only showed the relative abundance of mammalian virus-related sequences at the family and genus level to provide information for future research (Mokili et al., 2012; Holmes, 2019). In addition, the relative abundance might not be a true measure of viral abundance due to the amplification by using random PCR, and the efficiency of primers might bias it. During the conduct of this study, care was taken to limit contamination. For example, different instruments were used to dissect different animals and different organs; experiments on different animal species were conducted on different days; all reagents used in the experiments were new. Negative control was established for each experiment.

All of the urban rats were *Rattus spp*. and they were trapped in the same region, which may account for the high degrees of similarity in the viral compositions among them (Halanova et al., 2018). We found a higher similarity in viral composition between SD rats and urban rats than between BALB/C mice and urban rats. The animal species might explain this result, SD rats belong to the animal species of *R. norvegicus* (Subramanian et al., 2007). Higher viral diversity was found in urban rats than in laboratory animals, which might be explained by the different living environments of these animals. The use of the same sampling region to trap urban rats and house shrews might be why there was a high degree of similarity between the results from these animals. *P. abramus* mainly feeds on insects and usually lives in residential areas, which leads to more opportunities for them to contact humans and animals living in urban environments (Li, 2011). This might account for the high degrees of similarity in viral composition among *P. abramus*, urban rats, and house shrews. It seems that the feeding habits, living environment, and animal species can all affect the viral communities in animals. Surprisingly, although *C. sphinx* (fruit-eating bats) (Corlett, 2011) and *H. larvatus* (insect-eating bats) (Syaripuddin et al., 2014) were trapped in different places, their viral composition was highly similar. This suggests that the type of animal, rather than their feeding habits or living environment, also affects viral composition in the liver.

In our study, sequences detected in the liver tissue samples from urban rats were related to the largest number of mammalian viruses. A previous study showed that the total number of zoonotic viruses in rodents is higher than in bats (Luis et al., 2013). The above indicated that more attention should be paid to urban rats to prevent transmission of emerging zoonotic viruses. Sequences related to potential etiologies of hepatitis, like herpesviruses, influenza A virus, HEV, TTV, and pegivirus, were detected in the liver tissue samples by using viral metagenomics.

To figure out the tropism of the viruses, we compared the virome in the serum and liver tissue samples from *R. norvegicus*. Sequences related to some viruses, such as herpesviruses, did have a higher relative abundance in liver tissue samples than serum samples, suggesting the liver tropism. However, sequences related to hepacivirus were only found in serum samples. In addition, sequences related to pegiviruses had a higher relative abundance in serum samples than liver tissue samples. Interestingly, sequences related to rhinovirus C, a cause of acute respiratory tract infections, were only detected in the liver tissue samples (Erkkola et al., 2020). However, the relationship between it and liver diseases is still elusive. Attention should be paid to the viruses that have liver tropism to understand the etiologies of hepatitis. In addition, viral communities between liver tissue and serum samples from the same animals showed high differences, indicating that the serum remaining in liver tissue samples during dissection might have little impact on the results of liver tissue samples.

To investigate the prevalence and genetic diversity of some animal viruses related to etiologies and potential etiologies of human hepatitis, fifteen viruses were detected in the trapped animals by using PCR methods. However, sequences related to hepacivirus were only detected by using viral metagenomics. The discrepancy between the results of viral metagenomics and PCR can be explained by some reasons. On the one hand, viral metagenomics is more sensitive than PCR, and it is an efficient method for viral discovery. On the other hand, there might be false-positive results in viral metagenomics due to short reads. Using PCR methods, we found sequences related to hepatovirus, influenza A virus, herpesviruses, adenovirus, ZIKV, pegivirus, and TTV in these animals.

The first detection of sequences related to hepatovirus in *H. larvatus* was reported in our study. In addition, we also reported the first detection of sequences related to ZIKV in urban rats and house shrews. However, these sequences were only detected by using PCR methods, not by viral metagenomics, which might be explained by the small sample sizes in viral metagenomics. Bat hepatovirus sequences obtained in our study were highly similar to one hepatovirus sequence from *Hipposideros armiger* at both the nucleotide and amino acid levels, suggesting that the hepatovirus sequences obtained from *H. larvatus* and *H. armiger* belonged to the same species of hepatovirus (Zell et al., 2017), and cross-species transmission of hepatovirus had happened between bats. Sequences related to ZIKV detected in different species of animals clustered together, suggesting the potential of cross-species transmission of it.

Using the PCR method, sequences related to influenza A virus were only detected in liver tissue samples. The detection rates were 2.97%, 2.88%, and 43.50% in bats, urban rats, and house shrews, respectively. A previous study also reported the influenza A virus matrix gene segments in rats (Cummings et al., 2019). Although the sequences related to influenza A virus obtained from viral metagenomics showed a high degree of similarity with the neuraminidase gene and nucleocapsid protein gene sequences of influenza A virus, a relatively low similarity was found between the PCR-screened sequences and the matrix gene sequences of influenza A virus from GenBank. The matrix gene contributes to the host restriction of influenza A virus (Calderon et al., 2019). It seems that the sequences related to influenza A virus in these animals might belong to two new viral species within the genus *Alphainfluenzavirus*, and these viruses may be host-specific. However, sequences related to the matrix gene of influenza A virus in urban rats and house shrews were similar, suggesting that the viruses related to influenza A virus in these animals might share a common ancestor. None of the serum samples was positive in the detection of sequences related to influenza A virus by using PCR, which indicated that viruses related to influenza A virus in these animals may be hepatotropic. Previous studies showed that the influenza A virus can cause liver injury in human and laboratory rats (Morocco et al., 2014; Nonaka et al., 2019; Zhang et al., 2019). These indicated that like the human influenza A virus, the viruses related to the influenza A virus in animals might also be a causative agent of hepatitis. Influenza viruses from other animals, like swine, have been confirmed to be zoonotic viruses (Honce and Schultz-Cherry, 2020). Still, the zoonotic potential of the viruses related to influenza A virus in bats, urban rats, and shrews is unknown. More research is needed to amplify longer genomic segments of these viruses, investigate the pathogenicity and zoonotic potential, and provide some information for disease prevention.

Similar to sequences related to influenza A virus, sequences related to animal herpesviruses were also only found in liver tissue samples from bats, urban rats, and house shrews by using the PCR method, with the detection rates of 20.79%, 21.96%, and 2.26%, respectively. Using the metagenomic sequencing method, potential viral sequences related to human herpesviruses were reported. In addition, the relative abundance of sequences related to herpesviruses in liver tissue samples was significantly higher than in serum samples (Supplementary Figures 4, 5). These results not only indicate that bats, urban rats, and house shrews are animal reservoirs for herpesviruses, but further suggest the hepatotropism of herpesviruses in these animals, which, similar to herpesviruses in human, might also cause hepatitis (Suto et al., 1995). It is worth noting that high relative abundances of sequences related to herpesviruses were also found in laboratory rats and mice (Supplementary Figure 2). Herpesviruses might have a symbiotic relationship with bats, rats, mice, and shrews, and are closely related to the survival and evolution of these animals. In performing experiments on animals in the future, therefore, care should be taken to pay attention to whether the herpesviruses have any effect on the results.

Sequence related to adenovirus was only detected in one liver tissue sample from rats by using PCR method. None of the serum samples was positive for it by using PCR or viral metagenomics. It seems that rats, bats and shrews may not be the natural hosts of adenovirus. However, a previous study reported higher detection rates of adenovirus sequences in the fecal samples from rats and shrews (Zheng et al., 2016). Difference in the results between our study and the previous study might be explained by the sample type. Rats, bats, and shrews may not be susceptible to adenoviruses. We still need more large-scale studies to confirm the host range of this virus.

Detection of pegivirus sequences in urban rats and house shrews indicated that these animals were hosts for pegivirus. All of the pegivirus sequences obtained in our study belonged to *pegivirus J* (rodent pegivirus). They showed high similarity with each other, which suggested cross-species transmission of pegivirus among these animals. No suitable small animal model for pegivirus exists (Bailey et al., 2015). Minus-strand pegivirus RNA was only detected in *R. norvegicus*, which indicated the replication of pegivirus in *R. norvegicus*, and therefore, they might be a suitable animal model for the investigation of pegivirus. In addition, more attention should be paid to *R. norvegicus* for the prevention of the transmission of pegivirus. However, pegivirus had a higher detection rate in the serum samples than liver tissue samples by using the PCR method. Besides, sequences related to pegiviruses also had higher relative abundance in the serum samples than the liver tissue samples using viral metagenomics (Supplementary Figure 5). In addition, minus-strand pegivirus RNA was found in serum samples from six *R. norvegicus* but not in their liver tissue samples. The evidence indicated that rodent pegivirus might have a wide tropism. Further research, such as cell culture, real-time PCR, and animal model, is required to better understand in the tropism and pathogenicity of it (George et al., 2006; Liu et al., 2016).

TTV sequences were detected in urban rats and house shrews, with significantly higher detection rates in urban rats. This suggested that urban rats are common hosts for TTV. Sequences from the same species of animals were usually clustered together. TTV might be a species-specific virus, but the cross-species transmission of it was also found between urban rats in our study. Consistent with the results of a previous study on KIs-V, TTV sequences were also detected at a significantly higher rate in HEV-positive animals, highlighting the similar mechanisms of host cell entry between TTV and HEV (Lole et al., 2017; He et al., 2018). In addition, a previous study showed that TTV only replicates in the liver (Hu et al., 2002). It seems that TTV may have a relationship with liver diseases, and we still need more experiments to figure out whether it is an etiology of hepatitis or not.

Our research provides some basic results that further understand the liver virome in bats, urban rats, and house shrews. We also demonstrated the wide circulation of a diverse set of sequences related to the potential causes of hepatitis in these animals. The presented findings form a framework for future investigations of the etiologies of hepatitis and provide an insight into the monitoring of emerging zoonotic viruses in small animals. However, our study still had some limitations. Firstly, we did not identify the age and sex of the animals, which might affect the viral composition within them. Secondly, the sample size used for the viral metagenomic analysis was small, so our metagenomic results should be confirmed by more rigorous studies with larger sample sizes. Thirdly, strict standards should be set to consider genuine virus-related sequences in future research, including the length and identities of the sequences and the number of the reads (Cantalupo and Pipas, 2019; Gebremedhn et al., 2020). Fourthly, not all the viral sequences obtained from viral metagenomics were verified by confirmatory PCR tests. PCR tests should be used to detect these virus-related sequences in the future. In addition, the primers used in confirmatory PCR tests were not designed based on the sequences from viral metagenomics. The negative PCR results may be related to the mismatches across the primer binding regions or the false-positive results of metagenomic sequencing due to short reads. Fifthly, due a lack of pathologic descriptions of liver samples, we could not confirm the pathogenicity of the related viruses in animals. The pathogenicity of these viruses is still needed to be confirmed by animal experiments. Lastly, when performing viral metagenomic sequencing in the future, we should detect the viruses in all reagents to control reagent contamination.

## Conclusion

This is the first study to reveal and compare the viral community composition of liver tissue samples from bats, urban rats, mice, and house shrews. In addition, we also compared the viral community composition of serum samples and liver tissue samples from *R. norvegicus*. Our results showed differences in the viral composition of liver tissue samples between these animals, expanding our understanding of the virome within them. This study forms a framework for future investigations of the etiologies of hepatitis and provides an insight into the monitoring of emerging zoonotic viruses in small animals. The first detection of sequences related to the ZIKV in urban rats and house shrews was reported. Viruses related to influenza A virus and herpesviruses in urban rats and house shrews are both hepatotropic. *R. norvegicus* is a suitable animal model for pegivirus. TTV is hepatotropic, and it may have a relationship with liver diseases.

## Data Availability Statement

The datasets presented in this study can be found in online repositories. The representative genomes of the viruses presented in the study are deposited in the NCBI repository, accession numbers: MW055869-MW055884 and MW389532-MW389537. The viral metagenomic data presented in the study are deposited in the NCBI SRA repository, BioProject numbers: PRJNA695121 and PRJNA701687.

## Ethics Statement

The animal study was reviewed and approved by Animal Ethics and Welfare Committee of the School of Public Health, Southern Medical University.

## Author Contributions

WH and QC conceived of the project and QC obtained the funding. WH contributed to the writing of the manuscript. WH, YG, YW, and ZO performed the experiment. WH analyzed the data. WH, XK, YL, and HH collected the samples. All of the authors have read and approved the manuscript for publication. All authors contributed to the article and approved the submitted version.

## Conflict of Interest

The authors declare that the research was conducted in the absence of any commercial or financial relationships that could be construed as a potential conflict of interest.

## Footnotes

1 https://github.com/najoshi/sickle

2 https://www.ncbi.nlm.nih.gov/nucleotide

3 https://www.ncbi.nlm.nih.gov/

4 https://www.nbisweden.github.io/MrBayes/

## Supplementary Material

Supplementary Figure 1 Relative abundance of the sequences related to known viruses in liver tissue samples from different animals.

Supplementary Figure 2 Relative abundance of the sequences related to mammalian viruses (family level) in liver tissue samples from different animals. Sequences with relative abundance less than 1% are not showed.

Supplementary Figure 3 Relative abundance of the sequences related to mammalian viruses (genus level) in liver tissue samples from different animals. Sequences with relative abundance less than 1% are not showed.

Supplementary Figure 4 Relative abundance of the sequences related to mammalian viruses (family level) in the serum and liver samples from *R. norvegicus*. Sequences with relative abundance less than 1% are not showed.

Supplementary Figure 5 Relative abundance of the sequences related to mammalian viruses (genus level) in the serum and liver samples from *R. norvegicus*. Sequences with relative abundance less than 1% are not showed.

Supplementary Figure 6 Phylogenetic tree constructed based on potential viral nucleotide sequence (100 bp, polyprotein gene) related to hepacivirus that obtained by using metagenomic sequencing (MrBayes, GTR + G + I nucleotide substitution model). A total of 29 representative sequences belonging to different species within genus *Hepacivirus* are included for comparison. One sequence belonging to genus *Flavivirus* is set as outgroup. Percentages of the posterior probability (PP) values are indicated.
