# Supplementary figures and images for: Detection of Virus-Related Sequences Associated With Potential Etiologies of Hepatitis in Liver Tissue Samples From Rats, Mice, Shrews, and Bats
Source: Front Microbiol. 2021 Jun 8;12:653873. doi: 10.3389/fmicb.2021.653873 (PMC8221242; doi:10.3389/fmicb.2021.653873)

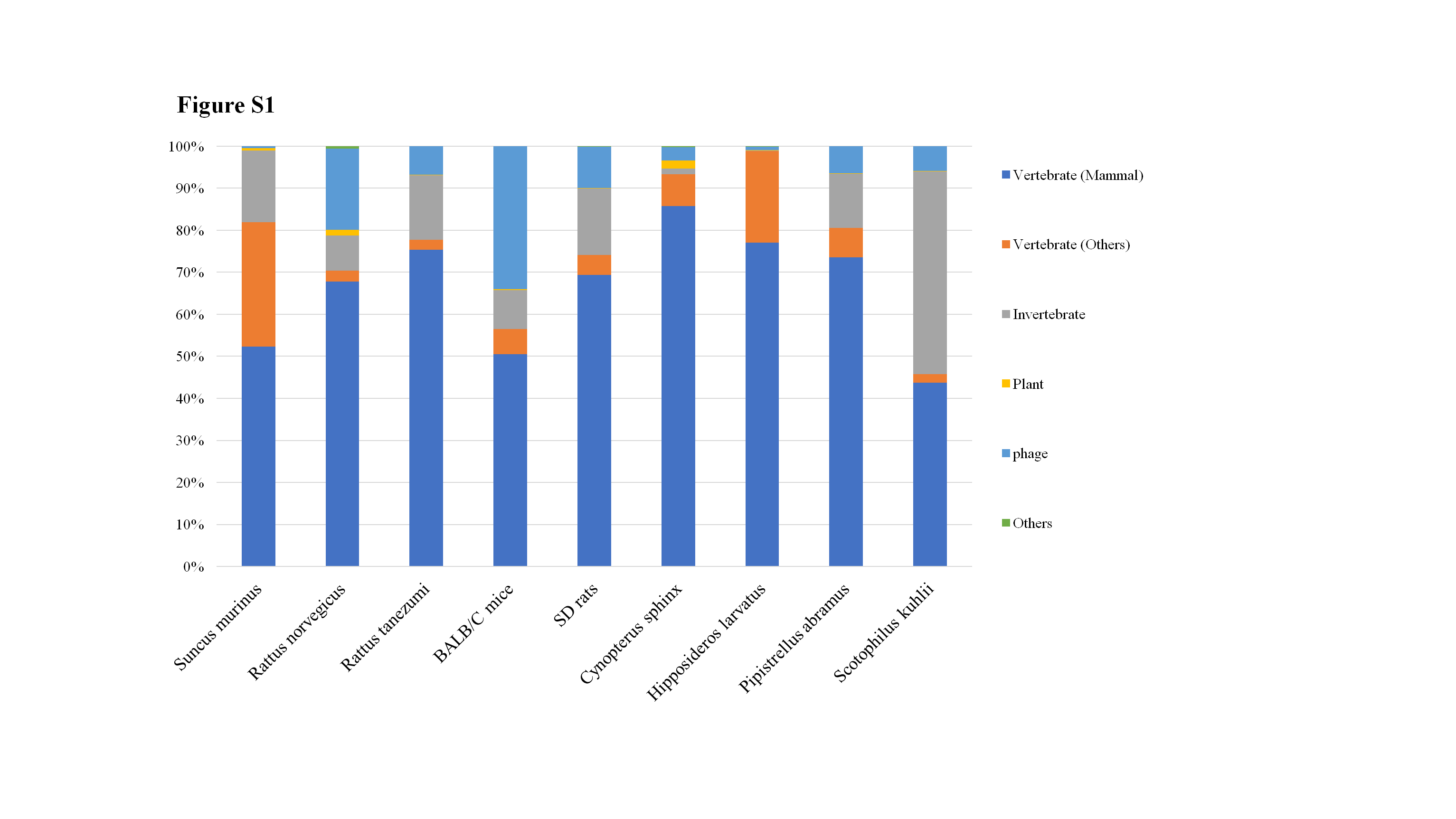

Supplement: Supplementary Figure 1 — Relative abundance of the sequences related to known viruses in liver tissue samples from different animals. [file Image_1.TIF]

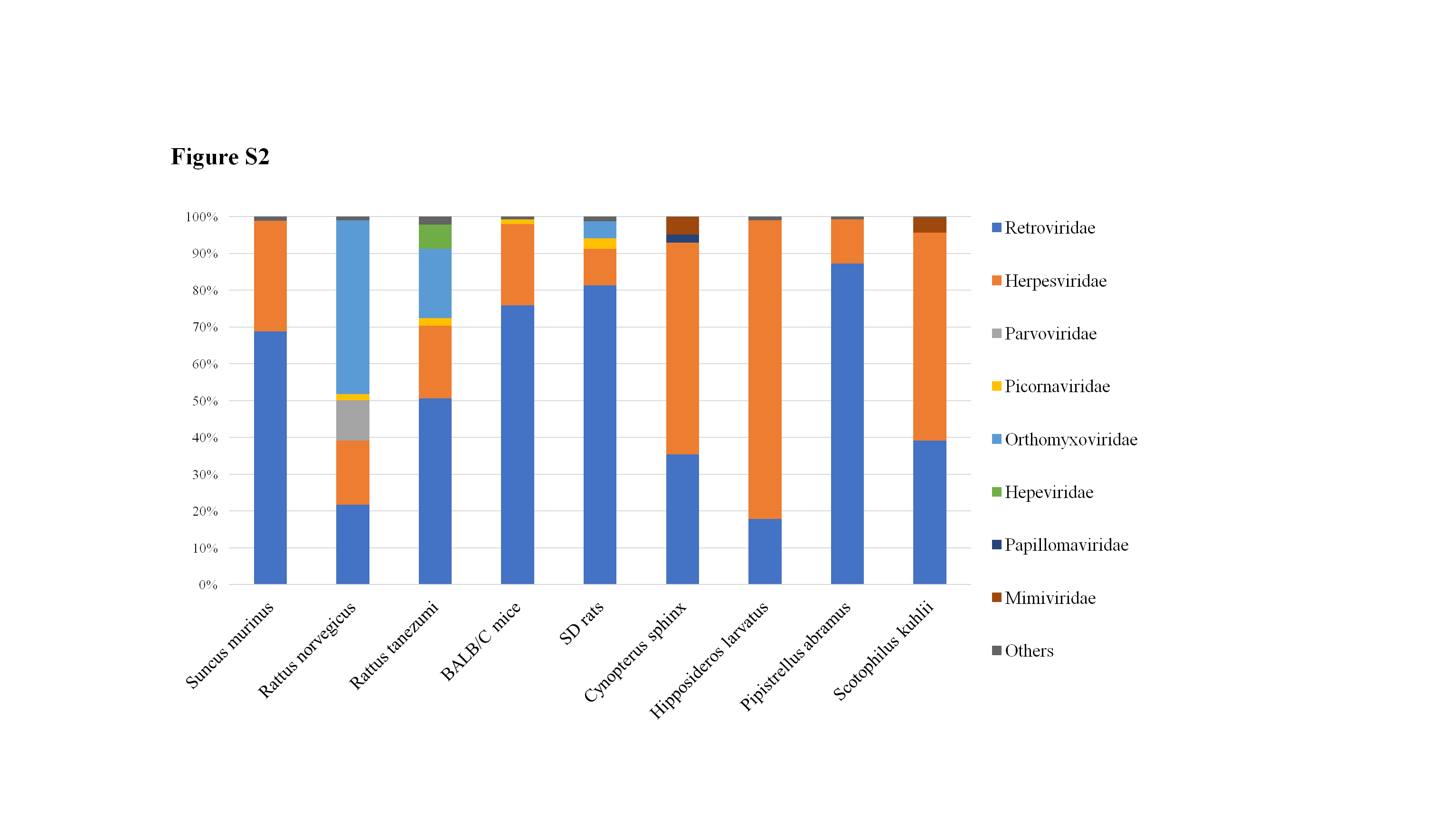

Supplement: Supplementary Figure 2 — Relative abundance of the sequences related to mammalian viruses (family level) in liver tissue samples from different animals. Sequences with relative abundance less than 1% are not showed. [file Image_2.TIF]

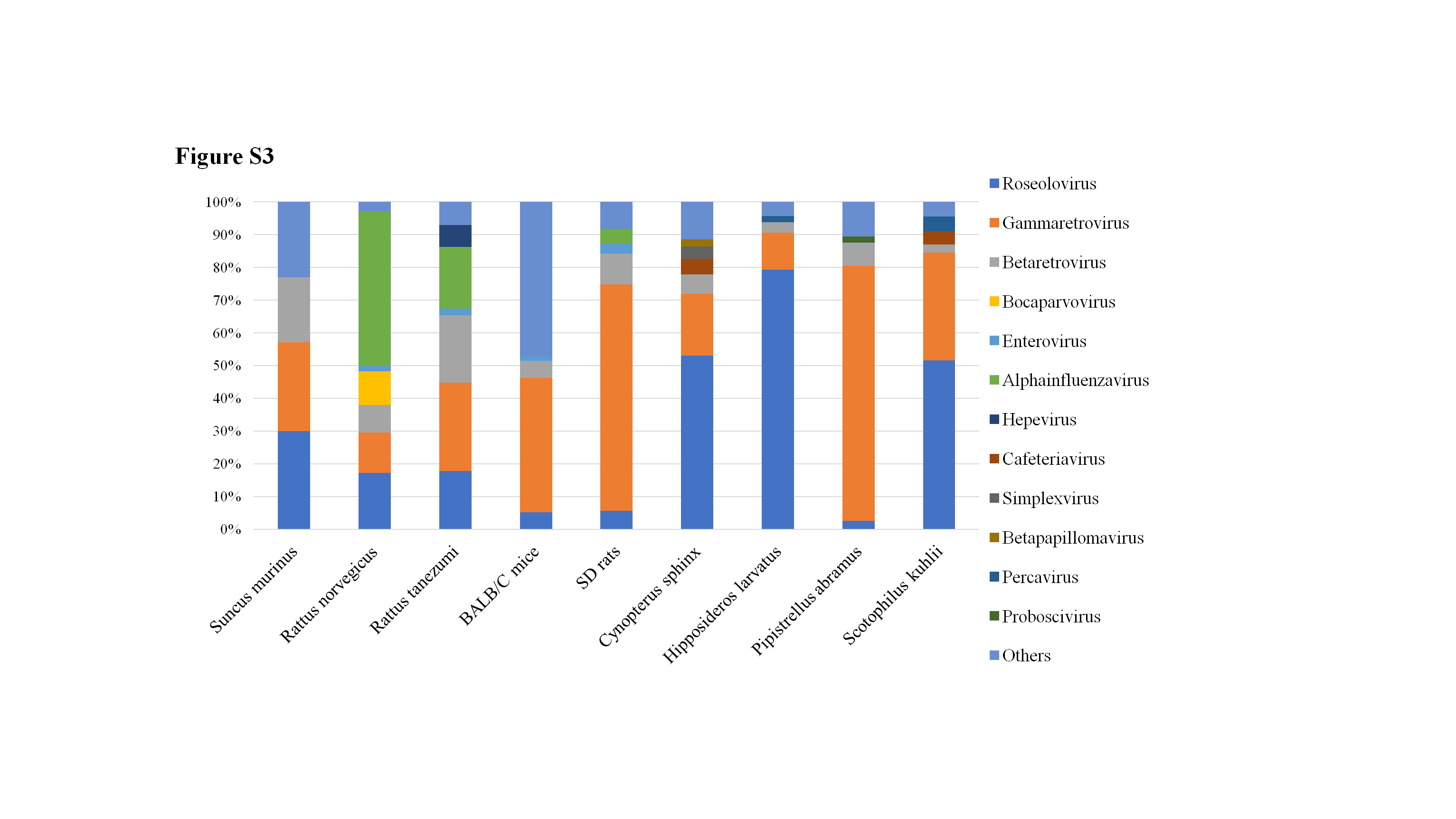

Supplement: Supplementary Figure 3 — Relative abundance of the sequences related to mammalian viruses (genus level) in liver tissue samples from different animals. Sequences with relative abundance less than 1% are not showed. [file Image_3.TIF]

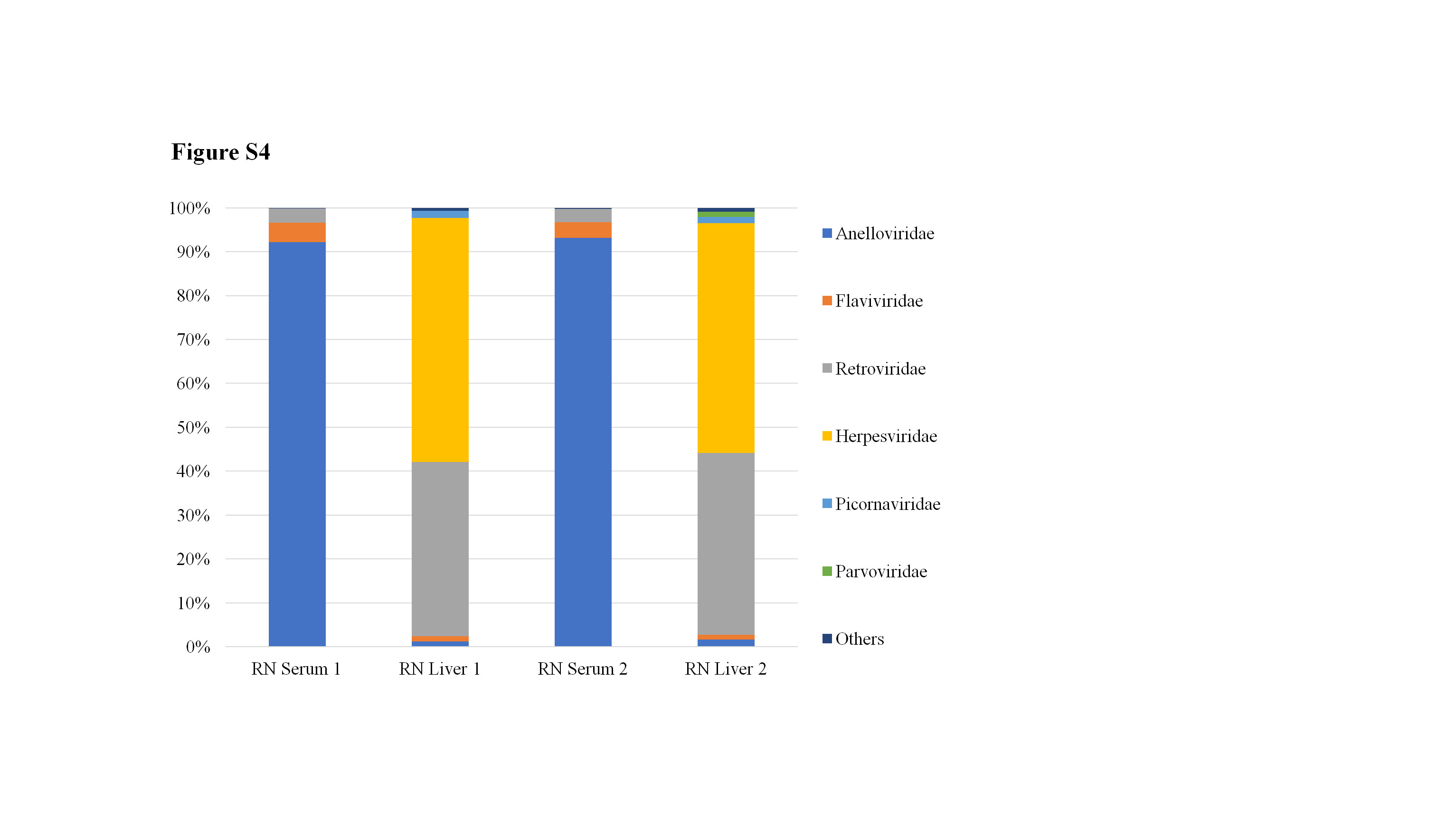

Supplement: Supplementary Figure 4 — Relative abundance of the sequences related to mammalian viruses (family level) in the serum and liver samples from R. norvegicus. Sequences with relative abundance less than 1% are not showed. [file Image_4.TIF]

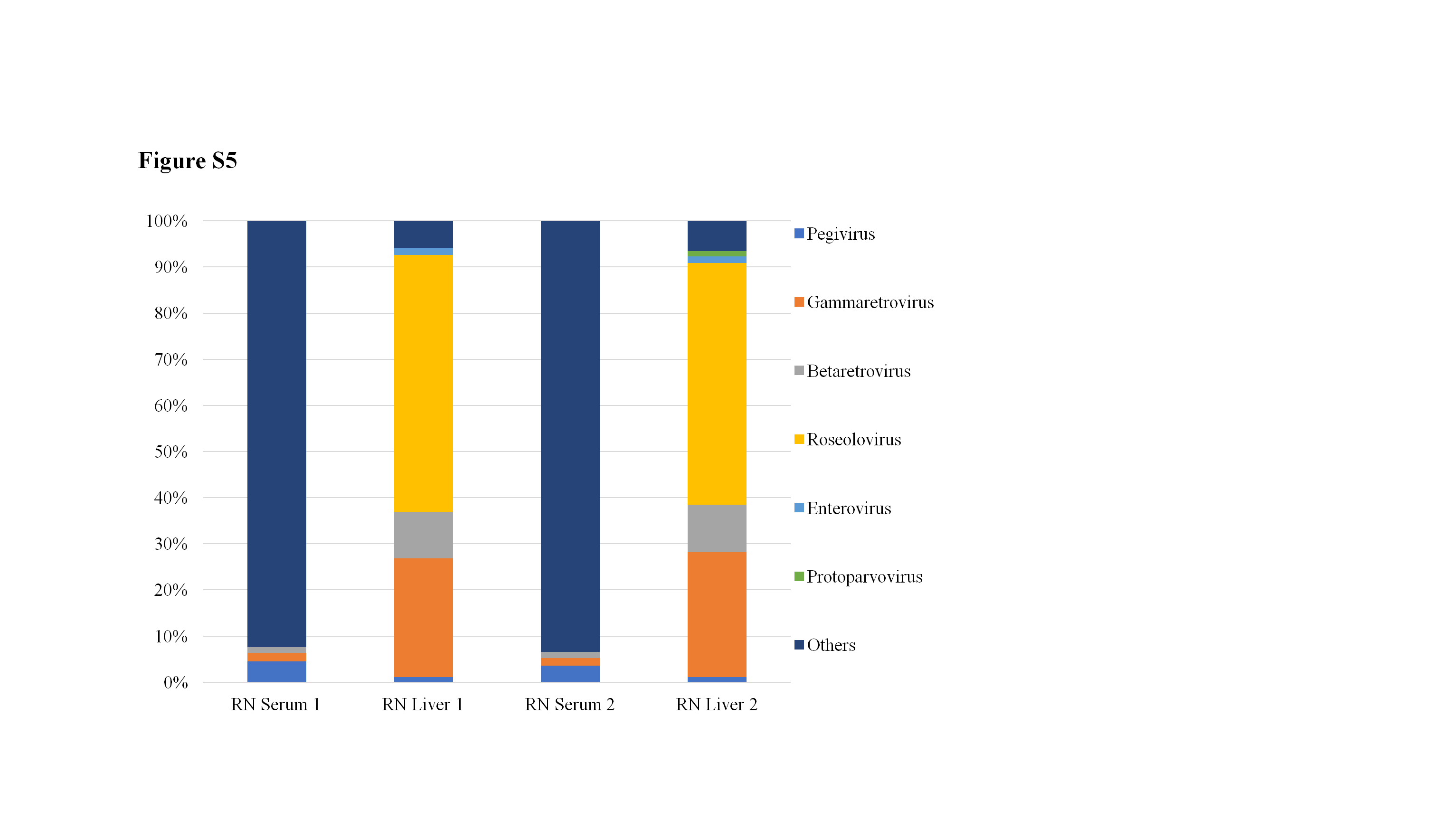

Supplement: Supplementary Figure 5 — Relative abundance of the sequences related to mammalian viruses (genus level) in the serum and liver samples from R. norvegicus. Sequences with relative abundance less than 1% are not showed. [file Image_5.TIF]

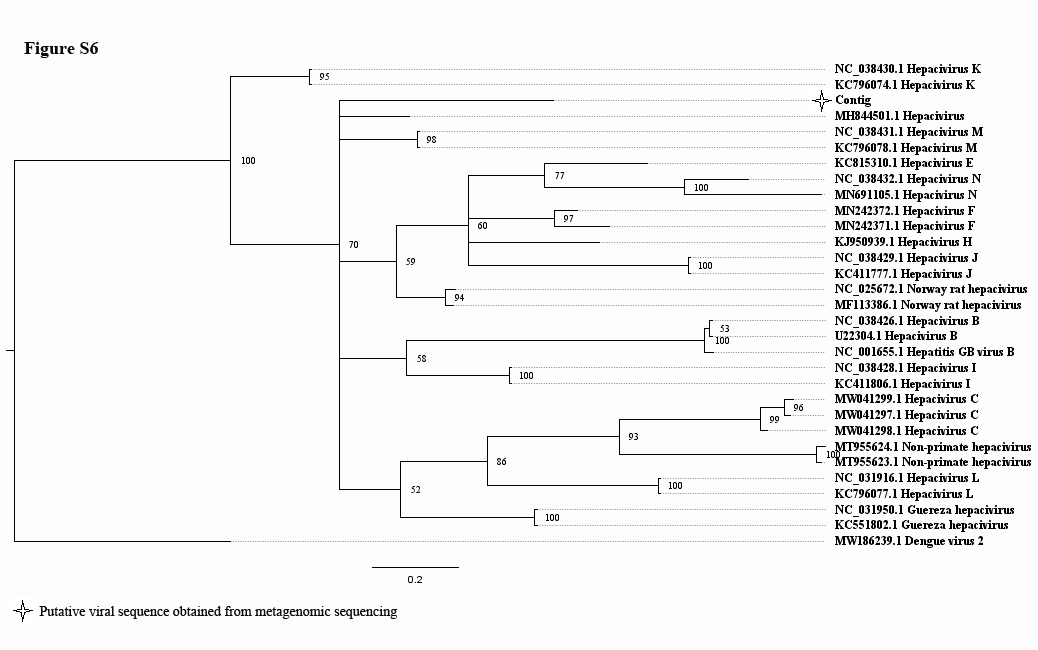

Supplement: Supplementary Figure 6 — Phylogenetic tree constructed based on potential viral nucleotide sequence (100 bp, polyprotein gene) related to hepacivirus that obtained by using metagenomic sequencing (MrBayes, GTR + G + I nucleotide substitution model). A total of 29 representative sequences belonging to different species within genus Hepacivirus are included for comparison. One sequence belonging to genus Flavivirus is set as outgroup. Percentages of the posterior probability (PP) values are indicated. [file Image_6.TIF]
