# Supplementary material for: Detection of Virus-Related Sequences Associated With Potential Etiologies of Hepatitis in Liver Tissue Samples From Rats, Mice, Shrews, and Bats
Source: Front Microbiol. 2021 Jun 8;12:653873. doi: 10.3389/fmicb.2021.653873 (PMC8221242; doi:10.3389/fmicb.2021.653873)
Supplement: Supplementary file 7 [file Table_1.DOCX]

Table S1.The summary of pooled samples for viral metagenomics

| Sample name | Group | Pool | Species | Sampling time | Trapped place |
| --- | --- | --- | --- | --- | --- |
| SMU177 | SM | 1 | *Suncus murinus* | 2015.06 | Guangzhou |
| SMU180 | SM | 1 | *Suncus murinus* | 2015.06 | Guangzhou |
| SMU188 | SM | 1 | *Suncus murinus* | 2015.06 | Guangzhou |
| SMU190 | SM | 1 | *Suncus murinus* | 2015.06 | Guangzhou |
| SMU210 | SM | 1 | *Suncus murinus* | 2015.06 | Guangzhou |
| SMU231 | SM | 1 | *Suncus murinus* | 2015.07 | Guangzhou |
| SMU236 | SM | 1 | *Suncus murinus* | 2015.07 | Guangzhou |
| SMU273 | SM | 1 | *Suncus murinus* | 2015.08 | Guangzhou |
| SMU275 | SM | 1 | *Suncus murinus* | 2015.08 | Guangzhou |
| SMU279 | SM | 1 | *Suncus murinus* | 2015.08 | Guangzhou |
| SMU285 | SM | 1 | *Suncus murinus* | 2015.08 | Guangzhou |
| SMU423 | SM | 1 | *Suncus murinus* | 2015.12 | Guangzhou |
| SMU424 | SM | 1 | *Suncus murinus* | 2015.12 | Guangzhou |
| SMU439 | SM | 1 | *Suncus murinus* | 2016.01 | Guangzhou |
| SMU447 | SM | 1 | *Suncus murinus* | 2016.01 | Guangzhou |
| SMU448 | SM | 1 | *Suncus murinus* | 2016.01 | Guangzhou |
| SMU449 | SM | 1 | *Suncus murinus* | 2016.01 | Guangzhou |
| SMU450 | SM | 1 | *Suncus murinus* | 2016.01 | Guangzhou |
| SMU451 | SM | 1 | *Suncus murinus* | 2016.01 | Guangzhou |
| SMU453 | SM | 1 | *Suncus murinus* | 2016.01 | Guangzhou |
| SMU454 | SM | 1 | *Suncus murinus* | 2016.01 | Guangzhou |
| SMU474 | SM | 1 | *Suncus murinus* | 2016.02 | Guangzhou |
| SMU477 | SM | 1 | *Suncus murinus* | 2016.02 | Guangzhou |
| SMU597 | SM | 1 | *Suncus murinus* | 2016.06 | Guangzhou |
| SMU187 | RN | 2 | *Rattus norvegicus* | 2015.06 | Guangzhou |
| SMU195 | RN | 2 | *Rattus norvegicus* | 2015.06 | Guangzhou |
| SMU201 | RN | 2 | *Rattus norvegicus* | 2015.06 | Guangzhou |
| SMU213 | RN | 2 | *Rattus norvegicus* | 2015.07 | Guangzhou |
| SMU216 | RN | 2 | *Rattus norvegicus* | 2015.07 | Guangzhou |
| SMU225 | RN | 2 | *Rattus norvegicus* | 2015.07 | Guangzhou |
| SMU259 | RN | 2 | *Rattus norvegicus* | 2015.08 | Guangzhou |
| SMU264 | RN | 2 | *Rattus norvegicus* | 2015.08 | Guangzhou |
| SMU435 | RN | 2 | *Rattus norvegicus* | 2015.12 | Guangzhou |
| SMU457 | RN | 2 | *Rattus norvegicus* | 2016.01 | Guangzhou |
| SMU461 | RN | 2 | *Rattus norvegicus* | 2016.01 | Guangzhou |
| SMU463 | RN | 2 | *Rattus norvegicus* | 2016.01 | Guangzhou |
| SMU482 | RN | 2 | *Rattus norvegicus* | 2016.02 | Guangzhou |
| SMU484 | RN | 2 | *Rattus norvegicus* | 2016.02 | Guangzhou |
| SMU485 | RN | 2 | *Rattus norvegicus* | 2016.02 | Guangzhou |
| SMU487 | RN | 2 | *Rattus norvegicus* | 2016.02 | Guangzhou |
| SMU489 | RN | 2 | *Rattus norvegicus* | 2016.02 | Guangzhou |
| SMU492 | RN | 2 | *Rattus norvegicus* | 2016.02 | Guangzhou |
| SMU493 | RN | 2 | *Rattus norvegicus* | 2016.02 | Guangzhou |
| SMU495 | RN | 2 | *Rattus norvegicus* | 2016.02 | Guangzhou |
| SMU591 | RN | 2 | *Rattus norvegicus* | 2016.06 | Guangzhou |
| SMU600 | RN | 2 | *Rattus norvegicus* | 2016.06 | Guangzhou |
| SMU605 | RN | 2 | *Rattus norvegicus* | 2016.06 | Guangzhou |
| SMU609 | RN | 2 | *Rattus norvegicus* | 2016.06 | Guangzhou |
| SMU430 | RT | 3 | *Rattus tanezumi* | 2015.12 | Guangzhou |
| SMU443 | RT | 3 | *Rattus tanezumi* | 2016.01 | Guangzhou |
| SMU480 | RT | 3 | *Rattus tanezumi* | 2016.02 | Guangzhou |
| SMU486 | RT | 3 | *Rattus tanezumi* | 2016.02 | Guangzhou |
| SMU490 | RT | 3 | *Rattus tanezumi* | 2016.02 | Guangzhou |
| HN2 | HL | 4 | *Hipposideros larvatus* | 2016.07 | Haikou |
| HN3 | HL | 4 | *Hipposideros larvatus* | 2016.07 | Haikou |
| HN4 | HL | 4 | *Hipposideros larvatus* | 2016.07 | Haikou |
| HN5 | HL | 4 | *Hipposideros larvatus* | 2016.07 | Haikou |
| HN6 | HL | 4 | *Hipposideros larvatus* | 2016.07 | Haikou |
| HN7 | HL | 4 | *Hipposideros larvatus* | 2016.07 | Haikou |
| HN8 | HL | 4 | *Hipposideros larvatus* | 2016.07 | Haikou |
| HN10 | HL | 4 | *Hipposideros larvatus* | 2016.07 | Haikou |
| HN11 | HL | 4 | *Hipposideros larvatus* | 2016.07 | Haikou |
| HN12 | HL | 4 | *Hipposideros larvatus* | 2016.07 | Haikou |
| HN13 | HL | 4 | *Hipposideros larvatus* | 2016.07 | Haikou |
| HN14 | HL | 4 | *Hipposideros larvatus* | 2016.07 | Haikou |
| MM71 | SK | 5 | *Scotophilus kuhlii* | 2016.07 | Maoming |
| MM72 | SK | 5 | *Scotophilus kuhlii* | 2016.07 | Maoming |
| MM73 | SK | 5 | *Scotophilus kuhlii* | 2016.07 | Maoming |
| MM74 | SK | 5 | *Scotophilus kuhlii* | 2016.07 | Maoming |
| MM75 | SK | 5 | *Scotophilus kuhlii* | 2016.07 | Maoming |
| MM76 | SK | 5 | *Scotophilus kuhlii* | 2016.07 | Maoming |
| MM77 | SK | 5 | *Scotophilus kuhlii* | 2016.07 | Maoming |
| MM78 | SK | 5 | *Scotophilus kuhlii* | 2016.07 | Maoming |
| MM79 | SK | 5 | *Scotophilus kuhlii* | 2016.07 | Maoming |
| MM80 | SK | 5 | *Scotophilus kuhlii* | 2016.07 | Maoming |
| MM81 | SK | 5 | *Scotophilus kuhlii* | 2016.07 | Maoming |
| MM82 | SK | 5 | *Scotophilus kuhlii* | 2016.07 | Maoming |
| GZ1 | PA | 6 | *Pipistrellus abramus* | 2015.11 | Guangzhou |
| GZ2 | PA | 6 | *Pipistrellus abramus* | 2015.11 | Guangzhou |
| GZ3 | PA | 6 | *Pipistrellus abramus* | 2015.11 | Guangzhou |
| GZ4 | PA | 6 | *Pipistrellus abramus* | 2015.11 | Guangzhou |
| GZ5 | PA | 6 | *Pipistrellus abramus* | 2015.11 | Guangzhou |
| GZ6 | PA | 6 | *Pipistrellus abramus* | 2015.11 | Guangzhou |
| GZ7 | CS | 7 | *Cynopterus sphinx* | 2015.11 | Guangzhou |
| GZ8 | CS | 7 | *Cynopterus sphinx* | 2015.11 | Guangzhou |
| GZ9 | CS | 7 | *Cynopterus sphinx* | 2015.11 | Guangzhou |
| GZ10 | CS | 7 | *Cynopterus sphinx* | 2015.11 | Guangzhou |
| GZ11 | CS | 7 | *Cynopterus sphinx* | 2015.11 | Guangzhou |
| GZ12 | CS | 7 | *Cynopterus sphinx* | 2015.11 | Guangzhou |
| GZ13 | CS | 7 | *Cynopterus sphinx* | 2015.11 | Guangzhou |
| GZ14 | CS | 7 | *Cynopterus sphinx* | 2015.11 | Guangzhou |
| GZ15 | CS | 7 | *Cynopterus sphinx* | 2015.11 | Guangzhou |
| GZ16 | CS | 7 | *Cynopterus sphinx* | 2015.11 | Guangzhou |
| GZ17 | CS | 7 | *Cynopterus sphinx* | 2015.11 | Guangzhou |
| GZ18 | CS | 7 | *Cynopterus sphinx* | 2015.11 | Guangzhou |
| BABL/C mouse 1 | BABL/C | 8 | BABL/C mouse | 2019.05 | Guangzhou |
| BABL/C mouse 2 | BABL/C | 8 | BABL/C mouse | 2019.05 | Guangzhou |
| BABL/C mouse 3 | BABL/C | 8 | BABL/C mouse | 2019.05 | Guangzhou |
| BABL/C mouse 4 | BABL/C | 8 | BABL/C mouse | 2019.05 | Guangzhou |
| BABL/C mouse 5 | BABL/C | 8 | BABL/C mouse | 2019.05 | Guangzhou |
| SD rat 1 | SD | 9 | SD rat | 2019.05 | Guangzhou |
| SD rat 2 | SD | 9 | SD rat | 2019.05 | Guangzhou |
| SD rat 3 | SD | 9 | SD rat | 2019.05 | Guangzhou |
| SD rat 4 | SD | 9 | SD rat | 2019.05 | Guangzhou |
| SD rat 5 | SD | 9 | SD rat | 2019.05 | Guangzhou |
| L32 | RN Liver 1 | 10 | *Rattus norvegicus* | 2018.05 | Xiamen |
| L34 | RN Liver 1 | 10 | *Rattus norvegicus* | 2018.05 | Xiamen |
| L38 | RN Liver 1 | 10 | *Rattus norvegicus* | 2018.05 | Xiamen |
| L39 | RN Liver 1 | 10 | *Rattus norvegicus* | 2018.05 | Xiamen |
| L47 | RN Liver 1 | 10 | *Rattus norvegicus* | 2018.05 | Xiamen |
| L66 | RN Liver 1 | 10 | *Rattus norvegicus* | 2018.05 | Xiamen |
| L71 | RN Liver 1 | 10 | *Rattus norvegicus* | 2018.05 | Xiamen |
| L76 | RN Liver 1 | 10 | *Rattus norvegicus* | 2018.05 | Xiamen |
| L96 | RN Liver 1 | 10 | *Rattus norvegicus* | 2018.05 | Xiamen |
| L100 | RN Liver 1 | 10 | *Rattus norvegicus* | 2018.05 | Xiamen |
| L102 | RN Liver 1 | 10 | *Rattus norvegicus* | 2018.05 | Xiamen |
| L107 | RN Liver 1 | 10 | *Rattus norvegicus* | 2018.05 | Xiamen |
| L111 | RN Liver 2 | 11 | *Rattus norvegicus* | 2018.05 | Xiamen |
| L127 | RN Liver 2 | 11 | *Rattus norvegicus* | 2018.05 | Xiamen |
| L135 | RN Liver 2 | 11 | *Rattus norvegicus* | 2018.05 | Xiamen |
| L139 | RN Liver 2 | 11 | *Rattus norvegicus* | 2018.05 | Xiamen |
| L152 | RN Liver 2 | 11 | *Rattus norvegicus* | 2018.05 | Xiamen |
| L154 | RN Liver 2 | 11 | *Rattus norvegicus* | 2018.05 | Xiamen |
| L155 | RN Liver 2 | 11 | *Rattus norvegicus* | 2018.05 | Xiamen |
| L157 | RN Liver 2 | 11 | *Rattus norvegicus* | 2018.05 | Xiamen |
| L161 | RN Liver 2 | 11 | *Rattus norvegicus* | 2018.05 | Xiamen |
| L166 | RN Liver 2 | 11 | *Rattus norvegicus* | 2018.05 | Xiamen |
| L167 | RN Liver 2 | 11 | *Rattus norvegicus* | 2018.05 | Xiamen |
| L169 | RN Liver 2 | 11 | *Rattus norvegicus* | 2018.05 | Xiamen |
| S32 | RN Serum 1 | 12 | *Rattus norvegicus* | 2018.05 | Xiamen |
| S34 | RN Serum 1 | 12 | *Rattus norvegicus* | 2018.05 | Xiamen |
| S38 | RN Serum 1 | 12 | *Rattus norvegicus* | 2018.05 | Xiamen |
| S39 | RN Serum 1 | 12 | *Rattus norvegicus* | 2018.05 | Xiamen |
| S47 | RN Serum 1 | 12 | *Rattus norvegicus* | 2018.05 | Xiamen |
| S66 | RN Serum 1 | 12 | *Rattus norvegicus* | 2018.05 | Xiamen |
| S71 | RN Serum 1 | 12 | *Rattus norvegicus* | 2018.05 | Xiamen |
| S76 | RN Serum 1 | 12 | *Rattus norvegicus* | 2018.05 | Xiamen |
| S96 | RN Serum 1 | 12 | *Rattus norvegicus* | 2018.05 | Xiamen |
| S100 | RN Serum 1 | 12 | *Rattus norvegicus* | 2018.05 | Xiamen |
| S102 | RN Serum 1 | 12 | *Rattus norvegicus* | 2018.05 | Xiamen |
| S107 | RN Serum 1 | 12 | *Rattus norvegicus* | 2018.05 | Xiamen |
| S111 | RN Serum 2 | 13 | *Rattus norvegicus* | 2018.05 | Xiamen |
| S127 | RN Serum 2 | 13 | *Rattus norvegicus* | 2018.05 | Xiamen |
| S135 | RN Serum 2 | 13 | *Rattus norvegicus* | 2018.05 | Xiamen |
| S139 | RN Serum 2 | 13 | *Rattus norvegicus* | 2018.05 | Xiamen |
| S152 | RN Serum 2 | 13 | *Rattus norvegicus* | 2018.05 | Xiamen |
| S154 | RN Serum 2 | 13 | *Rattus norvegicus* | 2018.05 | Xiamen |
| S155 | RN Serum 2 | 13 | *Rattus norvegicus* | 2018.05 | Xiamen |
| S157 | RN Serum 2 | 13 | *Rattus norvegicus* | 2018.05 | Xiamen |
| S161 | RN Serum 2 | 13 | *Rattus norvegicus* | 2018.05 | Xiamen |
| S166 | RN Serum 2 | 13 | *Rattus norvegicus* | 2018.05 | Xiamen |
| S167 | RN Serum 2 | 13 | *Rattus norvegicus* | 2018.05 | Xiamen |
| S169 | RN Serum 2 | 13 | *Rattus norvegicus* | 2018.05 | Xiamen |

Table S2. Primers for viral detection and amplification

|  | Primer | Orientation | Sequence (5'-3') | Target fragment (base pairs) |
| --- | --- | --- | --- | --- |
| LV  (screening) | LV-F | Sense | TCTAGTAGGGGCTGTACCCG | 172 (this study) |
|  | LV-R | Antisense | GCTGGCATTCAAGTAGCCG |  |
| Pegivirus | F1 | Sense (first/second round) | ATGCAGGTGTTACTTCTCTTAG | 1028 (this study) |
|  | R1028 | Antisense (second round) | CCAAGAGTCCTCTGCCTGRG |  |
|  | R1083 | Antisense (first round) | GCACAGMCTGAACGAAAGTC |  |
|  | F906 | Sense (first/second round) | CTGGTGTGGAGTCTTGGCK | 969 (this study) |
|  | R1874 | Antisense (second round) | CCAGCAGTTRATGAGRCCRC |  |
|  | R1963 | Antisense (first round) | GGTACCACAACCCGTCAGG |  |
|  | F1836 | Sense (first/second round) | GTCYAAGTACCGTGAKGGT | 1100 (this study) |
|  | R2935 | Antisense (second round) | GAAGTAAGCCARGACGCACA |  |
|  | R2945 | Antisense (first round) | CWACRCAGCGGAAGTAAGCCA |  |
|  | F2575 | Sense (first/second round) | TGGTTCGACGCTGCTTCTTT | 1005 (this study) |
|  | R3579 | Antisense (second round) | GAATCRTCATGCACAGCACG |  |
|  | R3735 | Antisense (first round) | ACCCAATGTCCTTGCGCAC |  |
|  | F3535 | Sense (first/second round) | GTGYTGTACGACCAYTGYG | 1047 (this study) |
|  | R4581 | Antisense (second round) | ATCGGCCGTAAGTTGCGTAG |  |
|  | R4639 | Antisense (first round) | CACTCATCGCAAATCACGAC |  |
|  | F4491 | Sense (first/second round) | GTACATGAAGCAGTTGACTGG | 1031 (this study) |
|  | R5521 | Antisense (second round) | CACCTTGCCAGCGAACGTC |  |
|  | R5641 | Antisense (first round) | CGTCGTGGTAAGAGGTGTC |  |
|  | F5482 | Sense (first/second round) | CACGTGTGCTTGGAATCG | 1012 (this study) |
|  | R6492 | Antisense (second round) | GCATGCTTAGCAACCGGTTC |  |
|  | R6574 | Antisense (first round) | GGACARTGAGCGTACCATRTC |  |
|  | F6368 | Sense (first/second round) | GAGTCACGCTTGCTAAYGCT | 1041 (this study) |
|  | R7408 | Antisense (second round) | CAAARSCCTTCTCCATCAG |  |
|  | R7552 | Antisense (first round) | GTTGGCTGTCGAGCCACTC |  |
|  | F7282 | Sense (first/second round) | CTTCCACAAGTACCAGATGAC |  |
|  | R8332 | Antisense (second round) | ATTGGYACACCRCTCCAGAC | 1051 (this study) |
|  | R8419 | Antisense (first round) | CBGTCACGTACACYTTGTC |  |
|  | F8227 | Sense | GACGGGTGAGGAGGAGCT | 886 (this study) |
|  | R9112 | Antisense | CGCACTTGTGGTCAACGTG |  |
|  | F9045 | Sense (first/second round) | GGTACTTGTGCGTCAGCTG | 899 (this study) |
|  | R9943 | Antisense (second round) | CGTGAGGCTCAGTTGGAAC |  |
|  | R9972 | Antisense (first round) | ATAGCAGAGCGGGTGTCAAC |  |
| Adenovirus | F6911 | Sense (first/second round) | CGCACTCGACGAGCCAG | 420 (this study) |
|  | R7330 | Antisense (second round) | GTCCAGGGCCATCTGTAAG |  |
|  | R7385 | Antisense (first round) | CTGGGTCAAAGTACGAGATG |  |

Table S3. The summary of metagenomic sequencing results of the liver tissue samples from different animals.

| Group | Species | Raw reads | Clean reads | Contigs | Mean length of the contigs | ORFs |
| --- | --- | --- | --- | --- | --- | --- |
| BALB/C | BALB/C mouse | 23330096 | 23120005 | 1058 | 482 | 906 |
| SD | SD rat | 10532186 | 10258129 | 8288 | 388 | 3322 |
| RN | *Rattus norvegicus* | 5424981 | 5304188 | 3918 | 358 | 2008 |
| RT | *Rattus tanezumi* | 25775872 | 25436649 | 89769 | 281 | 31683 |
| SM | *Suncus murinus* | 33826098 | 33555108 | 66942 | 486 | 26856 |
| CS | *Cynopterus sphinx* | 29630080 | 29450149 | 55645 | 389 | 21058 |
| HL | *Hipposideros larvatus* | 34708504 | 34409513 | 163699 | 483 | 63840 |
| PA | *Pipistrellus abramus* | 27443076 | 27231423 | 105831 | 436 | 36454 |
| SK | *Scotophilus kuhlii* | 24752370 | 24452215 | 92458 | 412 | 34583 |
| RN Liver 1 | *Rattus norvegicus* | 35959880 | 6545852 | 202477 | 344 | 68799 |
| RN Liver 2 | *Rattus norvegicus* | 37959802 | 5882574 | 263167 | 354 | 89119 |
| RN Serum 1 | *Rattus norvegicus* | 41065710 | 35437111 | 877 | 3164 | 1550 |
| RN Serum 2 | *Rattus norvegicus* | 37969772 | 32672225 | 756 | 3620 | 1654 |

Table S4. Sequences related to mammalian viruses in the liver tissue samples from different animals.

| Group | Species | Family | | Genus |
| --- | --- | --- | --- | --- |
| BALB/C | BALB/C mouse | 10 | 11 | |
| SD | SD rat | 14 | 18 | |
| RN | *Rattus norvegicus* | 12 | 19 | |
| RT | *Rattus tanezumi* | 17 | 26 | |
| SM | *Suncus murinus* | 12 | 18 | |
| CS | *Cynopterus sphinx* | 9 | 16 | |
| HL | *Hipposideros larvatus* | 9 | 17 | |
| PA | *Pipistrellus abramus* | 7 | 13 | |
| SK | *Scotophilus kuhlii* | 12 | 21 | |

Table S5. Alpha diversity indices of different groups.

| Group | Shannon | Simpson | Chao | ACE |
| --- | --- | --- | --- | --- |
| SM | 2.9885 | 0.8578 | 49.2500 | 50.5800 |
| RN | 3.0146 | 0.8092 | 54.4286 | 60.6896 |
| RT | 3.3883 | 0.8761 | 70.4286 | 69.7426 |
| BALB/C | 2.7355 | 0.7996 | 28.5000 | 28.5626 |
| SD | 2.5519 | 0.7206 | 59.0000 | 44.5778 |
| CS | 2.8627 | 0.7778 | 48.3333 | 46.9725 |
| HL | 1.8272 | 0.5883 | 43.2000 | 47.2603 |
| PA | 2.2373 | 0.6262 | 31.5000 | 31.7728 |
| SK | 2.1848 | 0.6825 | 62.5000 | 66.2521 |
| RN Serum1 | 1.1759 | 0.3228 | 29.0000 | 28.8433 |
| RN Liver1 | 2.4558 | 0.7257 | 54.5000 | 56.2004 |
| RN Serum2 | 1.0864 | 0.2879 | 28.2500 | 29.5207 |
| RN Liver2 | 2.5474 | 0.7428 | 41.2500 | 50.8894 |

Table S6. Sequences related to mammalian viruses in the serum and liver tissue samples from *Rattus norvegicus*.

| Group | Family | | Genus |
| --- | --- | --- | --- |
| RN Serum 1 | 11 | 16 | |
| RN Liver 1 | 12 | 14 | |
| RN Serum 2 | 11 | 14 | |
| RN Liver 2 | 11 | 13 | |

Table S7. Nucleotide (nt) identity of the metagenomic sequences related to hepacivirus and rhinovirus C.

|  | Region | Length (bp) | Identity |
| --- | --- | --- | --- |
| Sequences related to hepacivirus | polyprotein gene | 100 | 76 |
| Sequences related to rhinovirus C | polyprotein gene | 151 | 94 |

Table S8. Results of the detection of viral sequences by using viral metagenomics and PCR methods.

| Viruses | Viral metagenomics | PCR confirmatory test |
| --- | --- | --- |
| Hepatovirus | - | + |
| Hepacivirus | + | - |
| Hepatitis D virus | - | - |
| Influenza A virus | + | + |
| Adenoviruse | + | + |
| Herpesvirus | + | + |
| Parechovirus | - | - |
| Pegivirus | + | + |
| Torque teno virus | + | + |
| Zika virus | - | + |
| Dengue virus | - | - |
| Chikungunya virus | - | - |
| Ljungan virus | - | - |
| KIs-Virus | - | - |
| SEN-Virus | - | - |

+: Positive result on the detection of viral sequences

-: Negative result on the detection of viral sequences

Table S9. Nucleotide (nt) identity of the PCR-screened sequences of influenza A virus

|  | 1 | 2 | 3 | 4 | 5 | 6 | 7 | 8 | 9 | 10 | 11 | 12 | 13 | 14 | 15 | 16 | 17 | 18 | 19 | 20 | 21 | 22 |
| --- | --- | --- | --- | --- | --- | --- | --- | --- | --- | --- | --- | --- | --- | --- | --- | --- | --- | --- | --- | --- | --- | --- |
| 1.YY61^1^ | ID | 1.00 | 1.00 | 0.99 | 1.00 | 1.00 | 0.96 | 1.00 | 0.94 | 0.44 | 0.43 | 0.15 | 0.30 | 0.32 | 0.42 | 0.41 | 0.24 | 0.22 | 0.20 | 0.18 | 0.19 | 0.13 |
| 2.YY13^1^ | 1.00 | ID | 1.00 | 0.99 | 1.00 | 1.00 | 0.96 | 1.00 | 0.94 | 0.44 | 0.43 | 0.15 | 0.30 | 0.32 | 0.42 | 0.41 | 0.24 | 0.22 | 0.20 | 0.18 | 0.19 | 0.13 |
| 3.YY83^2^ | 1.00 | 1.00 | ID | 0.99 | 1.00 | 1.00 | 0.96 | 1.00 | 0.94 | 0.44 | 0.43 | 0.15 | 0.30 | 0.32 | 0.42 | 0.41 | 0.24 | 0.22 | 0.20 | 0.18 | 0.19 | 0.13 |
| 4.MM80 ^1^ | 0.99 | 0.99 | 0.99 | ID | 0.99 | 0.99 | 0.96 | 0.99 | 0.93 | 0.43 | 0.43 | 0.14 | 0.29 | 0.31 | 0.41 | 0.41 | 0.23 | 0.22 | 0.19 | 0.18 | 0.18 | 0.12 |
| 5.GZ213^1^ | 1.00 | 1.00 | 1.00 | 0.99 | ID | 1.00 | 0.96 | 1.00 | 0.94 | 0.44 | 0.43 | 0.15 | 0.30 | 0.32 | 0.42 | 0.41 | 0.24 | 0.22 | 0.20 | 0.18 | 0.19 | 0.13 |
| 6.GZ235^4^ | 1.00 | 1.00 | 1.00 | 0.99 | 1.00 | ID | 0.96 | 1.00 | 0.94 | 0.44 | 0.43 | 0.15 | 0.30 | 0.32 | 0.42 | 0.41 | 0.24 | 0.22 | 0.20 | 0.18 | 0.19 | 0.13 |
| 7.GZ2^4^ | 0.96 | 0.96 | 0.96 | 0.96 | 0.96 | 0.96 | ID | 0.96 | 0.94 | 0.44 | 0.43 | 0.15 | 0.28 | 0.31 | 0.42 | 0.40 | 0.24 | 0.22 | 0.20 | 0.18 | 0.18 | 0.12 |
| 8.XM9^1^ | 1.00 | 1.00 | 1.00 | 0.99 | 1.00 | 1.00 | 0.96 | ID | 0.94 | 0.44 | 0.43 | 0.15 | 0.30 | 0.32 | 0.42 | 0.41 | 0.24 | 0.22 | 0.20 | 0.18 | 0.19 | 0.13 |
| 9.XM19^3^ | 0.94 | 0.94 | 0.94 | 0.93 | 0.94 | 0.94 | 0.94 | 0.94 | ID | 0.46 | 0.43 | 0.15 | 0.29 | 0.31 | 0.41 | 0.40 | 0.23 | 0.22 | 0.22 | 0.20 | 0.17 | 0.12 |
| 10.HN63^5^ | 0.44 | 0.44 | 0.44 | 0.43 | 0.44 | 0.44 | 0.44 | 0.44 | 0.46 | ID | 0.80 | 0.08 | 0.22 | 0.24 | 0.31 | 0.29 | 0.21 | 0.19 | 0.15 | 0.16 | 0.13 | 0.13 |
| 11.HN65^6^ | 0.43 | 0.43 | 0.43 | 0.43 | 0.43 | 0.43 | 0.43 | 0.43 | 0.43 | 0.80 | ID | 0.08 | 0.23 | 0.24 | 0.31 | 0.30 | 0.20 | 0.22 | 0.14 | 0.16 | 0.14 | 0.15 |
| 12.MT628433.1 Dhorithogotovirus | 0.15 | 0.15 | 0.15 | 0.14 | 0.15 | 0.15 | 0.15 | 0.15 | 0.15 | 0.08 | 0.08 | ID | 0.20 | 0.22 | 0.20 | 0.19 | 0.21 | 0.18 | 0.26 | 0.28 | 0.27 | 0.41 |
| 13.CY125948.1 Influenza A virus (H18N11) | 0.30 | 0.30 | 0.30 | 0.29 | 0.30 | 0.30 | 0.28 | 0.30 | 0.29 | 0.22 | 0.23 | 0.20 | ID | 0.84 | 0.68 | 0.68 | 0.45 | 0.23 | 0.19 | 0.27 | 0.25 | 0.23 |
| 14.CY103895.1 Influenza A virus (H17N10) | 0.32 | 0.32 | 0.32 | 0.31 | 0.32 | 0.32 | 0.31 | 0.32 | 0.31 | 0.24 | 0.24 | 0.22 | 0.84 | ID | 0.67 | 0.69 | 0.43 | 0.23 | 0.22 | 0.26 | 0.27 | 0.24 |
| 15.MK340168.1 Influenza A virus (H1N2) | 0.42 | 0.42 | 0.42 | 0.41 | 0.42 | 0.42 | 0.42 | 0.42 | 0.41 | 0.31 | 0.31 | 0.20 | 0.68 | 0.67 | ID | 0.92 | 0.45 | 0.24 | 0.23 | 0.26 | 0.26 | 0.26 |
| 16.MT419636.1 Influenza A virus (H1N1) | 0.41 | 0.41 | 0.41 | 0.41 | 0.41 | 0.41 | 0.40 | 0.41 | 0.40 | 0.29 | 0.30 | 0.19 | 0.68 | 0.69 | 0.92 | ID | 0.45 | 0.23 | 0.22 | 0.25 | 0.26 | 0.25 |
| 17.MW651801.1 Influenza B virus | 0.24 | 0.24 | 0.24 | 0.23 | 0.24 | 0.24 | 0.24 | 0.24 | 0.23 | 0.21 | 0.20 | 0.21 | 0.45 | 0.43 | 0.45 | 0.45 | ID | 0.25 | 0.21 | 0.27 | 0.27 | 0.27 |
| 18.MN885653.1 Salmon isavirus | 0.22 | 0.22 | 0.22 | 0.22 | 0.22 | 0.22 | 0.22 | 0.22 | 0.22 | 0.19 | 0.22 | 0.18 | 0.23 | 0.23 | 0.24 | 0.23 | 0.25 | ID | 0.20 | 0.26 | 0.21 | 0.23 |
| 19.MT246274.1 Influenza D virus | 0.20 | 0.20 | 0.20 | 0.19 | 0.20 | 0.20 | 0.20 | 0.20 | 0.22 | 0.15 | 0.14 | 0.26 | 0.19 | 0.22 | 0.23 | 0.22 | 0.21 | 0.20 | ID | 0.51 | 0.23 | 0.31 |
| 20.MK050101.1 Influenza C virus | 0.18 | 0.18 | 0.18 | 0.18 | 0.18 | 0.18 | 0.18 | 0.18 | 0.20 | 0.16 | 0.16 | 0.28 | 0.27 | 0.26 | 0.26 | 0.25 | 0.27 | 0.26 | 0.51 | ID | 0.25 | 0.41 |
| 21.NC 052929.1 Johnston Atoll quaranjavirus | 0.19 | 0.19 | 0.19 | 0.18 | 0.19 | 0.19 | 0.18 | 0.19 | 0.17 | 0.13 | 0.14 | 0.27 | 0.25 | 0.27 | 0.26 | 0.26 | 0.27 | 0.21 | 0.23 | 0.25 | ID | 0.23 |
| 22.MT628451.1 Thogoto thogotovirus | 0.13 | 0.13 | 0.13 | 0.12 | 0.13 | 0.13 | 0.12 | 0.13 | 0.12 | 0.13 | 0.15 | 0.41 | 0.23 | 0.24 | 0.26 | 0.25 | 0.27 | 0.23 | 0.31 | 0.41 | 0.23 | ID |

^1^: *Rattus norvegicus*

^2^: *Rattus tanezumi*

^3^: *Rattus losea*

^4^: *Suncus murinus*

^5^: *Hipposideros larvatus*

^6^: *Rhinolophus pusillus*

Table S10. Amino acid (aa) identity of the PCR-screened sequences of influenza A virus

|  | 1 | 2 | 3 | 4 | 5 | 6 | 7 | 8 | 9 | 10 | 11 | 12 | 13 | 14 | 15 | 16 | 17 | 18 | 19 | 20 | 21 | 22 |
| --- | --- | --- | --- | --- | --- | --- | --- | --- | --- | --- | --- | --- | --- | --- | --- | --- | --- | --- | --- | --- | --- | --- |
| 1.YY61^1^ | ID | 1.00 | 1.00 | 0.98 | 1.00 | 1.00 | 0.92 | 1.00 | 0.88 | 0.22 | 0.22 | 0.01 | 0.12 | 0.12 | 0.28 | 0.25 | 0.08 | 0.01 | 0.03 | 0.02 | 0.04 | 0.03 |
| 2.YY13^1^ | 1.00 | ID | 1.00 | 0.98 | 1.00 | 1.00 | 0.92 | 1.00 | 0.88 | 0.22 | 0.22 | 0.01 | 0.12 | 0.12 | 0.28 | 0.25 | 0.08 | 0.01 | 0.03 | 0.02 | 0.04 | 0.03 |
| 3.YY83^2^ | 1.00 | 1.00 | ID | 0.98 | 1.00 | 1.00 | 0.92 | 1.00 | 0.88 | 0.22 | 0.22 | 0.01 | 0.12 | 0.12 | 0.28 | 0.25 | 0.08 | 0.01 | 0.03 | 0.02 | 0.04 | 0.03 |
| 4.MM80 ^1^ | 0.98 | 0.98 | 0.98 | ID | 0.98 | 0.98 | 0.90 | 0.98 | 0.85 | 0.22 | 0.22 | 0.01 | 0.10 | 0.10 | 0.27 | 0.23 | 0.08 | 0.01 | 0.03 | 0.02 | 0.04 | 0.03 |
| 5.GZ213^1^ | 1.00 | 1.00 | 1.00 | 0.98 | ID | 1.00 | 0.92 | 1.00 | 0.88 | 0.22 | 0.22 | 0.01 | 0.12 | 0.12 | 0.28 | 0.25 | 0.08 | 0.01 | 0.03 | 0.02 | 0.04 | 0.03 |
| 6.GZ235^4^ | 1.00 | 1.00 | 1.00 | 0.98 | 1.00 | ID | 0.92 | 1.00 | 0.88 | 0.22 | 0.22 | 0.01 | 0.12 | 0.12 | 0.28 | 0.25 | 0.08 | 0.01 | 0.03 | 0.02 | 0.04 | 0.03 |
| 7.GZ2^4^ | 0.92 | 0.92 | 0.92 | 0.90 | 0.92 | 0.92 | ID | 0.92 | 0.90 | 0.22 | 0.22 | 0.01 | 0.10 | 0.10 | 0.28 | 0.22 | 0.08 | 0.01 | 0.03 | 0.00 | 0.04 | 0.03 |
| 8.XM9^1^ | 1.00 | 1.00 | 1.00 | 0.98 | 1.00 | 1.00 | 0.92 | ID | 0.88 | 0.22 | 0.22 | 0.01 | 0.12 | 0.12 | 0.28 | 0.25 | 0.08 | 0.01 | 0.03 | 0.02 | 0.04 | 0.03 |
| 9.XM19^3^ | 0.88 | 0.88 | 0.88 | 0.85 | 0.88 | 0.88 | 0.90 | 0.88 | ID | 0.22 | 0.20 | 0.01 | 0.10 | 0.10 | 0.25 | 0.20 | 0.08 | 0.01 | 0.03 | 0.00 | 0.04 | 0.01 |
| 10.HN63^5^ | 0.22 | 0.22 | 0.22 | 0.22 | 0.22 | 0.22 | 0.22 | 0.22 | 0.22 | ID | 0.73 | 0.01 | 0.09 | 0.09 | 0.21 | 0.17 | 0.04 | 0.04 | 0.00 | 0.03 | 0.03 | 0.07 |
| 11.HN65^6^ | 0.22 | 0.22 | 0.22 | 0.22 | 0.22 | 0.22 | 0.22 | 0.22 | 0.20 | 0.73 | ID | 0.01 | 0.09 | 0.09 | 0.22 | 0.19 | 0.05 | 0.03 | 0.01 | 0.05 | 0.03 | 0.07 |
| 12.MT628433.1 Dhorithogotovirus | 0.01 | 0.01 | 0.01 | 0.01 | 0.01 | 0.01 | 0.01 | 0.01 | 0.01 | 0.01 | 0.01 | ID | 0.04 | 0.06 | 0.01 | 0.01 | 0.09 | 0.04 | 0.05 | 0.05 | 0.08 | 0.15 |
| 13.CY125948.1 Influenza A virus (H18N11) | 0.12 | 0.12 | 0.12 | 0.10 | 0.12 | 0.12 | 0.10 | 0.12 | 0.10 | 0.09 | 0.09 | 0.04 | ID | 0.62 | 0.30 | 0.31 | 0.23 | 0.06 | 0.05 | 0.08 | 0.07 | 0.06 |
| 14.CY103895.1 Influenza A virus (H17N10) | 0.12 | 0.12 | 0.12 | 0.10 | 0.12 | 0.12 | 0.10 | 0.12 | 0.10 | 0.09 | 0.09 | 0.06 | 0.62 | ID | 0.28 | 0.33 | 0.16 | 0.04 | 0.03 | 0.09 | 0.10 | 0.07 |
| 15.MK340168.1 Influenza A virus (H1N2) | 0.28 | 0.28 | 0.28 | 0.27 | 0.28 | 0.28 | 0.28 | 0.28 | 0.25 | 0.21 | 0.22 | 0.01 | 0.30 | 0.28 | ID | 0.80 | 0.16 | 0.04 | 0.08 | 0.08 | 0.06 | 0.09 |
| 16.MT419636.1 Influenza A virus (H1N1) | 0.25 | 0.25 | 0.25 | 0.23 | 0.25 | 0.25 | 0.22 | 0.25 | 0.20 | 0.17 | 0.19 | 0.01 | 0.31 | 0.33 | 0.80 | ID | 0.15 | 0.06 | 0.06 | 0.08 | 0.07 | 0.07 |
| 17.MW651801.1 Influenza B virus | 0.08 | 0.08 | 0.08 | 0.08 | 0.08 | 0.08 | 0.08 | 0.08 | 0.08 | 0.04 | 0.05 | 0.09 | 0.23 | 0.16 | 0.16 | 0.15 | ID | 0.04 | 0.03 | 0.03 | 0.11 | 0.10 |
| 18.MN885653.1 Salmon isavirus | 0.01 | 0.01 | 0.01 | 0.01 | 0.01 | 0.01 | 0.01 | 0.01 | 0.01 | 0.04 | 0.03 | 0.04 | 0.06 | 0.04 | 0.04 | 0.06 | 0.04 | ID | 0.01 | 0.07 | 0.03 | 0.08 |
| 19.MT246274.1 Influenza D virus | 0.03 | 0.03 | 0.03 | 0.03 | 0.03 | 0.03 | 0.03 | 0.03 | 0.03 | 0.00 | 0.01 | 0.05 | 0.05 | 0.03 | 0.08 | 0.06 | 0.03 | 0.01 | ID | 0.28 | 0.06 | 0.08 |
| 20.MK050101.1 Influenza C virus | 0.02 | 0.02 | 0.02 | 0.02 | 0.02 | 0.02 | 0.00 | 0.02 | 0.00 | 0.03 | 0.05 | 0.05 | 0.08 | 0.09 | 0.08 | 0.08 | 0.03 | 0.07 | 0.28 | ID | 0.06 | 0.15 |
| 21.NC 052929.1 Johnston Atoll quaranjavirus | 0.04 | 0.04 | 0.04 | 0.04 | 0.04 | 0.04 | 0.04 | 0.04 | 0.04 | 0.03 | 0.03 | 0.08 | 0.07 | 0.10 | 0.06 | 0.07 | 0.11 | 0.03 | 0.06 | 0.06 | ID | 0.03 |
| 22.MT628451.1 Thogoto thogotovirus | 0.03 | 0.03 | 0.03 | 0.03 | 0.03 | 0.03 | 0.03 | 0.03 | 0.01 | 0.07 | 0.07 | 0.15 | 0.06 | 0.07 | 0.09 | 0.07 | 0.10 | 0.08 | 0.08 | 0.15 | 0.03 | ID |

^1^: *Rattus norvegicus*

^2^: *Rattus tanezumi*

^3^: *Rattus losea*

^4^: *Suncus murinus*

^5^: *Hipposideros larvatus*

^6^: *Rhinolophus pusillus*

Table S11. Nucleotide (nt) and amino acid identity of the PCR-screened sequences of Zika virus.

|  | GZ454 | GZ458 | GZ462 | GZ474 | GZ483 | GZ52 | MK028860.1 | MN185328.1 | NC_012532.1 | MN190155.1 | MT078741.1 | MK566202.1 | MK696551.1 | AY858048.2 |
| --- | --- | --- | --- | --- | --- | --- | --- | --- | --- | --- | --- | --- | --- | --- |
| GZ454^2^ | ID | 1.000 (1.000) | 0.987 (0.961) | 1.000 (1.000) | 0.993 (0.980) | 0.993 (1.000) | 0.849 (0.826) | 0.955 (0.903) | 0.836 (0.826) | 0.962 (0.903) | 0.962 (0.903) | 0.962 (0.903) | 0.962 (0.903) | 0.641 (0.653) |
| GZ458^1^ | 1.000 (1.000) | ID | 0.987 (0.961) | 1.000 (1.000) | 0.993 (0.980) | 0.993 (1.000) | 0.849 (0.826) | 0.955 (0.903) | 0.836 (0.826) | 0.962 (0.903) | 0.962 (0.903) | 0.962 (0.903) | 0.962 (0.903) | 0.641 (0.653) |
| GZ462^1^ | 0.987 (0.961) | 0.987 (0.961) | ID | 0.987 (0.961) | 0.981 (0.942) | 0.981 (0.961) | 0.836 (0.788) | 0.943 (0.865) | 0.823 (0.788) | 0.949 (0.865) | 0.949 (0.865) | 0.949 (0.865) | 0.949 (0.865) | 0.635 (0.653) |
| GZ474^2^ | 1.000 (1.000) | 1.000 (1.000) | 0.987 (0.961) | ID | 0.993 (0.980) | 0.993 (1.000) | 0.849 (0.826) | 0.955  (0.903) | 0.836 (0.826) | 0.962 (0.903) | 0.962 (0.903) | 0.962 (0.903) | 0.962 (0.903) | 0.641 (0.653) |
| GZ483^1^ | 0.993 (0.980) | 0.993 (0.980) | 0.981 (0.942) | 0.993 (0.980) | ID | 0.987 (0.980) | 0.849 (0.826) | 0.949 (0.903) | 0.830 (0.826) | 0.955 (0.903) | 0.955 (0.903) | 0.955 (0.903) | 0.955 (0.903) | 0.647 (0.653) |
| GZ52^1^ | 0.993 (1.000) | 0.993 (1.000) | 0.981 (0.961) | 0.993 (1.000) | 0.987 (0.980) | ID | 0.849 (0.826) | 0.955 (0.903) | 0.836 (0.826) | 0.962 (0.903) | 0.962 (0.903) | 0.962 (0.903) | 0.962 (0.903) | 0.641 (0.653) |
| MK028860.1 | 0.849 (0.826) | 0.849 (0.826) | 0.836 (0.788) | 0.849 (0.826) | 0.849 (0.826) | 0.849 (0.826) | ID | 0.879 (0.923) | 0.917 (1.000) | 0.886 (0.923) | 0.886 (0.923) | 0.886 (0.923) | 0.886 (0.923) | 0.708 (0.730) |
| MN185328.1 | 0.955 (0.903) | 0.955 (0.903) | 0.943 (0.865) | 0.955 (0.903) | 0.949 (0.903) | 0.955 (0.903) | 0.879 (0.923) | ID | 0.867 (0.923) | 0.993 (1.000) | 0.993 (1.000) | 0.993 (1.000) | 0.993 (1.000) | 0.664 (0.750) |
| NC_012532.1 | 0.836 (0.826) | 0.836 (0.826) | 0.823 (0.788) | 0.836 (0.826) | 0.830 (0.826) | 0.836 (0.826) | 0.917 (1.000) | 0.867 (0.923) | ID | 0.873 (0.923) | 0.873 (0.923) | 0.873 (0.923) | 0.873 (0.923) | 0.696 (0.730) |
| MN190155.1 | 0.962 (0.903) | 0.962 (0.903) | 0.949 (0.865) | 0.962 (0.903) | 0.955 (0.903) | 0.962 (0.903) | 0.886 (0.923) | 0.993 (1.000) | 0.873 (0.923) | ID | 1.000 (1.000) | 1.000 (1.000) | 1.000 (1.000) | 0.670 (0.750) |
| MT078741.1 | 0.962 (0.903) | 0.962 (0.903) | 0.949 (0.865) | 0.962 (0.903) | 0.955 (0.903) | 0.962 (0.903) | 0.886 (0.923) | 0.993 (1.000) | 0.873 (0.923) | 1.000 (1.000) | ID | 1.000 (1.000) | 1.000 (1.000) | 0.670 (0.750) |
| MK566202.1 | 0.962 (0.903) | 0.962 (0.903) | 0.949 (0.865) | 0.962 (0.903) | 0.955 (0.903) | 0.962 (0.903) | 0.886 (0.923) | 0.993 (1.000) | 0.873 (0.923) | 1.000 (1.000) | 1.000 (1.000) | ID | 1.000 (1.000) | 0.670 (0.750) |
| MK696551.1 | 0.962 (0.903) | 0.962 (0.903) | 0.949 (0.865) | 0.962 (0.903) | 0.955 (0.903) | 0.962 (0.903) | 0.886 (0.923) | 0.993 (1.000) | 0.873 (0.923) | 1.000 (1.000) | 1.000 (1.000) | 1.000 (1.000) | ID | 0.670 (0.750) |
| AY858048.2 | 0.641 (0.653) | 0.641 (0.653) | 0.635 (0.653) | 0.641 (0.653) | 0.647 (0.653) | 0.641 (0.653) | 0.708 (0.730) | 0.664 (0.750) | 0.696 (0.730) | 0.670 (0.750) | 0.670 (0.750) | 0.670 (0.750) | 0.670 (0.750) | ID |

MK028860.1, MN185328.1, NC_012532.1, MN190155.1, MT078741.1, MK566202.1, MK696551.1: GenBank accesson number of Zika virus sequences

AY858048.2: GenBank accesson number of dengue virus sequence

Amino acid identity are showed in brackets

^1^: *Rattus norvegicus*

^2^: *Suncus murinus*

Table S12. Nucleotide (nt) and amino acid identity of the near-full-length pegivirus genomes.

|  | Nt identity | | | | | | | | | | | |  | | | Amimo acid idenity | | | | | | | | | | |
| --- | --- | --- | --- | --- | --- | --- | --- | --- | --- | --- | --- | --- | --- | --- | --- | --- | --- | --- | --- | --- | --- | --- | --- | --- | --- | --- |
|  | MH179063 | | | SMU461 | | MM87 | | XM50 | | YN33 | MG273686 | | | MH179063 | | | SMU461 | | | MM87 | | XM50 | | YN33 | | MG273686 |
| MH179063 | | ID | 0.432 | | 0.433 | | 0.431 | | 0.432 | | | 0.432 | | | ID | | | 0.278 | 0.277 | | 0.274 | | 0.278 | | 0.279 | |
| SMU461 | | 0.432 | ID | | 0.944 | | 0.941 | | 0.948 | | | 0.959 | | | 0.278 | | | ID | 0.984 | | 0.956 | | 0.986 | | 0.986 | |
| MM87 | | 0.433 | 0.944 | | ID | | 0.936 | | 0.942 | | | 0.941 | | | 0.277 | | | 0.984 | ID | | 0.956 | | 0.985 | | 0.982 | |
| XM50 | | 0.431 | 0.941 | | 0.936 | | ID | | 0.939 | | | 0.939 | | | 0.274 | | | 0.956 | 0.956 | | ID | | 0.959 | | 0.955 | |
| YN33 | | 0.432 | 0.948 | | 0.942 | | 0.939 | | ID | | | 0.946 | | | 0.278 | | | 0.986 | 0.985 | | 0.959 | | ID | | 0.985 | |
| MG273686 | | 0.432 | 0.959 | | 0.941 | | 0.939 | | 0.946 | | | ID | | | 0.279 | | | 0.986 | 0.982 | | 0.955 | | 0.985 | | ID | |

MH179063: GenBank accesson number of pegivirus sequence from human.

MG273686: GenBank accesson number of pegivirus sequence from rat.

Table S13. Nucleotide acid identity of the near-full-length TTV genomes.

|  | LC381845 | YY61^1^ | YN29^1^ | XM49^1^ | GZ28^1^ | GZ56^1^ | MM87^1^ | GZHD56^2^ | XM113^2^ | XM85^3^ | GZ441^4^ | MF926282 |
| --- | --- | --- | --- | --- | --- | --- | --- | --- | --- | --- | --- | --- |

| LC381845 | ID | 0.330 | 0.328 | 0.335 | 0.339 | 0.335 | 0.333 | 0.320 | 0.326 | 0.331 | 0.270 | 0.330 |
| --- | --- | --- | --- | --- | --- | --- | --- | --- | --- | --- | --- | --- |
| YY61^1^ | 0.330 | ID | 0.958 | 0.932 | 0.927 | 0.942 | 0.946 | 0.730 | 0.739 | 0.952 | 0.676 | 1.000 |
| YN29^1^ | 0.328 | 0.958 | ID | 0.927 | 0.921 | 0.944 | 0.947 | 0.729 | 0.736 | 0.948 | 0.671 | 0.958 |
| XM49^1^ | 0.335 | 0.932 | 0.927 | ID | 0.967 | 0.959 | 0.912 | 0.730 | 0.739 | 0.925 | 0.668 | 0.932 |
| GZ28^1^ | 0.339 | 0.927 | 0.921 | 0.967 | ID | 0.960 | 0.920 | 0.735 | 0.745 | 0.927 | 0.672 | 0.927 |
| GZ56^1^ | 0.335 | 0.942 | 0.944 | 0.959 | 0.960 | ID | 0.923 | 0.727 | 0.741 | 0.928 | 0.674 | 0.942 |
| MM87^1^ | 0.333 | 0.946 | 0.947 | 0.912 | 0.920 | 0.923 | ID | 0.730 | 0.736 | 0.943 | 0.673 | 0.946 |
| GZHD56^2^ | 0.320 | 0.730 | 0.729 | 0.730 | 0.735 | 0.727 | 0.730 | ID | 0.877 | 0.727 | 0.595 | 0.73 |
| XM113^2^ | 0.326 | 0.739 | 0.736 | 0.739 | 0.745 | 0.741 | 0.736 | 0.877 | ID | 0.734 | 0.611 | 0.739 |
| XM85^3^ | 0.331 | 0.952 | 0.948 | 0.925 | 0.927 | 0.928 | 0.943 | 0.727 | 0.734 | ID | 0.666 | 0.952 |
| GZ441^4^ | 0.270 | 0.676 | 0.671 | 0.668 | 0.672 | 0.674 | 0.673 | 0.595 | 0.611 | 0.666 | ID | 0.676 |
| MF926282 | 0.330 | 1.000 | 0.958 | 0.932 | 0.927 | 0.942 | 0.946 | 0.730 | 0.739 | 0.952 | 0.676 | ID |

LC381845: GenBank accesson number of TTV sequence from human.

MF926282: GenBank accesson number of TTV sequence from rat.

^1^: *Rattus norvegicus*

^2^: *Rattus tanezumi*

^3^: *Rattus losea*

^4^: *Suncus murinus*

Table S14. Amino acid identity of the near-full-length TTV genomes.

|  | LC381845 | | YY61^1^ | | YN29^1^ | | XM49^1^ | | GZ28^1^ | | GZ56^1^ | | MM87^1^ | GZHD56^2^ | XM113^2^ | XM85^3^ | GZ441^4^ | MF926282 |
| --- | --- | --- | --- | --- | --- | --- | --- | --- | --- | --- | --- | --- | --- | --- | --- | --- | --- | --- |
| LC381845 | | ID | | 0.175 | | 0.179 | | 0.176 | | 0.181 | | 0.178 | 0.180 | 0.159 | 0.164 | 0.175 | 0.162 | 0.175 |
| YY61^1^ | | 0.175 | | ID | | 0.922 | | 0.940 | | 0.874 | | 0.951 | 0.886 | 0.626 | 0.638 | 0.892 | 0.677 | 1.000 |
| YN29^1^ | | 0.179 | | 0.922 | | ID | | 0.904 | | 0.878 | | 0.915 | 0.897 | 0.627 | 0.652 | 0.899 | 0.676 | 0.922 |
| XM49^1^ | | 0.176 | | 0.940 | | 0.904 | | ID | | 0.901 | | 0.962 | 0.856 | 0.624 | 0.640 | 0.871 | 0.675 | 0.940 |
| GZ28^1^ | | 0.181 | | 0.874 | | 0.878 | | 0.901 | | ID | | 0.896 | 0.904 | 0.654 | 0.651 | 0.936 | 0.671 | 0.874 |
| GZ56^1^ | | 0.178 | | 0.951 | | 0.915 | | 0.962 | | 0.896 | | ID | 0.861 | 0.618 | 0.635 | 0.882 | 0.669 | 0.951 |
| MM87^1^ | | 0.180 | | 0.886 | | 0.897 | | 0.856 | | 0.904 | | 0.861 | ID | 0.636 | 0.656 | 0.918 | 0.675 | 0.886 |
| GZHD56^2^ | | 0.159 | | 0.626 | | 0.627 | | 0.624 | | 0.654 | | 0.618 | 0.636 | ID | 0.854 | 0.648 | 0.572 | 0.626 |
| XM113^2^ | | 0.164 | | 0.638 | | 0.652 | | 0.640 | | 0.651 | | 0.635 | 0.656 | 0.854 | ID | 0.641 | 0.583 | 0.638 |
| XM85^3^ | | 0.175 | | 0.892 | | 0.899 | | 0.871 | | 0.936 | | 0.882 | 0.918 | 0.648 | 0.641 | ID | 0.669 | 0.892 |
| GZ441^4^ | | 0.162 | | 0.677 | | 0.676 | | 0.675 | | 0.671 | | 0.669 | 0.675 | 0.572 | 0.583 | 0.669 | ID | 0.677 |
| MF926282 | | 0.175 | | 1.000 | | 0.922 | | 0.940 | | 0.874 | | 0.951 | 0.886 | 0.626 | 0.638 | 0.892 | 0.677 | ID |

LC381845: GenBank accesson number of TTV sequence from human.

MF926282: GenBank accesson number of TTV sequence from rat.

^1^: *Rattus norvegicus*

^2^: *Rattus tanezumi*

^3^: *Rattus losea*

^4^: *Suncus murinus*
